# Quantitative genomics-enabled selection for simultaneous improvement of lint yield and seed traits in cotton (*Gossypium hirsutum* L.)

**DOI:** 10.1007/s00122-024-04645-6

**Published:** 2024-05-26

**Authors:** Zitong Li, Qian-Hao Zhu, Philippe Moncuquet, Iain Wilson, Danny Llewellyn, Warwick Stiller, Shiming Liu

**Affiliations:** 1 CSIRO Agriculture and Food, Canberra, ACT 2601 Australia; 2 CSIRO Agriculture and Food, Narrabri, NSW 2390 Australia

## Abstract

**Key message:**

A Bayesian linkage disequilibrium-based multiple-locus mixed model identified QTLs for fibre, seed and oil traits and predicted breeding worthiness of test lines, enabling their simultaneous improvement in cotton.

**Abstract:**

Improving cotton seed and oil yields has become increasingly important while continuing to breed for higher lint yield. In this study, a novel Bayesian linkage disequilibrium-based multiple-locus mixed model was developed for QTL identification and genomic prediction (GP). A multi-parent population consisting of 256 recombinant inbred lines, derived from four elite cultivars with distinct combinations of traits, was used in the analysis of QTLs for lint percentage, seed index, lint index and seed oil content and their interrelations. All four traits were moderately heritable and correlated but with no large influence of genotype × environment interactions across multiple seasons. Seven to ten major QTLs were identified for each trait with many being adjacent or overlapping for different trait pairs. A fivefold cross-validation of the model indicated prediction accuracies of 0.46–0.62. GP results based on any two-season phenotypes were strongly correlated with phenotypic means of a pooled analysis of three-season experiments (*r* = 0.83–0.92). When used for selection of improvement in lint, seed and oil yields, GP captured 40–100% of individuals with comparable lint yields of those selected based on the three-season phenotypic results. Thus, this quantitative genomics-enabled approach can not only decipher the genomic variation underlying lint, seed and seed oil traits and their interrelations, but can provide predictions for their simultaneous improvement. We discuss future breeding strategies in cotton that will enhance the entire value of the crop, not just its fibre.

**Supplementary Information:**

The online version contains supplementary material available at 10.1007/s00122-024-04645-6.

## Introduction

Cotton is primarily produced to supply natural fibres for the textile industry, and its seeds are often just a by-product. Over the five years from 2015 to 2019, the world produced about 73 million tonnes of seed cotton annually that ends up in 24.3 million tonnes of lint fibre and 42.5 million tonnes of cottonseeds after ginning (https://www.fao.org/faostat/en/#data/QCL). The much higher market value of the lint contributes most to the economic returns from growing cotton (≈ 80%) (Zeng et al. 2015), but the relatively high oil and protein content of cottonseed also incentivises diversified end uses and adds extra value to production. Oil is the most valuable part of crushed cottonseeds and can be used in cooking, food ingredient, industrial lubricants or as biofuels, and makes cotton one of the important oil crops in the world, while the cottonseeds can also be directly used to feed ruminant livestock (Liu et al. 2009).

Driven by the current value proposition, genetic improvement in cotton has long been focused on increasing lint productivity and, where possible, better fibre quality but often at the expense of seed size and quality, that has in some cases resulted in lower seed establishment rates and seedling vigour (Maeda et al. 2023). While that trend may continue, there is a renewed interest in concurrently improving cottonseed oil yield and oil quality (Liu et al. 2009; Campbell et al. 2016; Maeda et al. 2023). The aims are to increase or at least maintain oil content in cottonseeds while breeding for higher lint yield to meet the needs of post-harvest processors but also cotton growers who require high quality planting seeds. There are also efforts to alter the composition of cottonseed oil (by genetic manipulation or gene editing) to improve seed germination at low temperatures (Gao et al. 2020) or to make cotton seeds more nutritional as a food for animals or even humans and make cotton oil comparable in quality to oils from other crops including canola and soybean (Liu et al. 2009; Wu et al. 2022). To facilitate this, there is a need to better understand heritable variation and relationships of yield and seed component traits in cotton.

Lint percentage (LP), seed index (SI) and lint index (LI) are three foundational yield components (traits) in cotton (Worley et al. 1974). Lint and seed yields are the outcomes of LI or SI multiplied by the total number of harvestable seeds in the field. Thus, yield improvement can be achieved by producing more seeds per unit area while maintaining other yield variables or vice versa or both (Ruan 2013; Liu et al. 2020b). Significant genetic variation for cotton yield and seed component traits is present in existing *G. hirsutum* germplasm collections (Kohel 1978), elite breeding lines (Dowd et al. 2018; Liu et al. 2015b; Zeng et al. 2015; Zhao et al. 2019; Wang et al. 2021; Zhu et al. 2021), and within purposely created populations used for quantitative and molecular genetic studies (Campbell et al. 2016; Liu et al. 2015a; Kothari et al. 2016; Wang et al. 2019; Yu et al. 2012; Yuan et al. 2018; Li et al. 2023). When the genetics underlying variation for those traits was dissected, additive effects were found to be more important than dominance and other interaction effects and notable maternal effects were also reported for SI and seed oil content (SOC) (Wu et al. 2010). The narrow sense heritability reported ranged from 0.33 to 0.81 for LP (Campbell and Myers 2015; Li et al. 2023), 0.80 for LI (Ali and Awan 2009), and from 0.12 to 0.52 for SOC (Khan et al. 2007; Kothari et al. 2016). Therefore, they are all moderately to highly heritable such that crossing breeding and phenotypic selection are an effective approach for improving seed yield component traits.

Since the development of molecular markers, extensive research has been conducted to identify genomic regions associated with phenotypic variations of yield related traits in cotton. The studies used either natural populations (Liu et al. 2015b; Fang et al. 2017; Yuan et al. 2018; Ma et al. 2018; Zhao et al. 2019; Zhu et al. 2021; Wang et al. 2021) or purposely developed linkage mapping populations. The latter included bi-parental crosses-derived recombinant inbred lines (RILs) and backcross-derived recombinant inbred lines (BILs) (Liu et al. 2015a; Shang et al. 2016; Wang et al. 2019; Yu et al. 2012; Yuan et al. 2018; Li et al. 2023). A large number of quantitative trait loci (QTLs) have been found for individual traits. For example, Wang et al. (2007) identified four QTLs for LP, three for LI and two for SI in an intraspecific *G. hirsutum* RIL population, but only one LP QTL was detected across different test environments explaining 13.4% phenotypic variance (PV). Using two RIL and backcross populations in *G. hirsutum*, Shang et al. (2016) identified 9 and 18 stable QTLs for SOC and SI, respectively, and further dissected additive effects of single-locus and epistatic interactions between loci important for both SI and SOC in *G. hirsutum*. The majority of QTLs for LP, SI, LI and SOC reported so far only account for a small proportion of PVs (< 15%), and their presence also depends on test environments, type and density of molecular makers, and types of population and sample sizes used in the studies. However, on some occasions, stable and major QTLs were reported; for example, Wang et al. (2019) using density SLAF-seq markers and *G. hirsutum* RIL populations, found 1 to 4 stable QTLs for SI, kernel weight and kernel oil and protein content that accounted for at least 40% of PV. Furthermore, several refined regions or candidate genes, such as *GhSI7* for seed index (Liu et al. 2022), *GhPRXR1* (Ma et al. 2019) and *qOil-3* for seed oil content (Liu et al. 2020a), were fine mapped and functionally analysed, with flanking markers now available for breeding selection. To increase genetic diversity and also power to identify genomic regions and/or genes associated with traits, Multi-parent Advanced Generation Intercross (MAGIC) populations in cotton have been developed and used for QTL discovery for fibre quality and yield traits (Thyssen et al. 2019; Li et al. 2023), but not yet for cotton yield related and seed oil traits.

In plant breeding (including cotton) research, the majority of genome-wide association and QTL mapping has been conducted based on single-locus mapping methods (Brachi et al. 2011; Khan et al. 2021; Zhao et al. 2019). Those single-locus approaches used a marginal linear model to separately estimate the association between phenotypes and each individual SNP. Then the *p*-values from the analyses were adjusted in a multiple testing procedure such as using Bonferroni correction and permutation tests (Joo et al. 2016). For a natural population potentially with cryptic relatedness among samples, a random effect or principal components (PC) estimate based on genetic data needs to be added into the regression model to control false positive findings (Khan et al. 2021). In contrast, multiple-locus methods have also been proposed to simultaneously link multiple SNPs to phenotypes (Segura et al. 2012; Miao et al. 2018; Cortes et al. 2021). There are multiple benefits of using multiple-locus methods. First, a multiple-locus method can provide estimation of effects of any SNP conditionally on all other SNPs. This may be helpful to control false positives as well as improve the power to detect true QTL (Cortes et al. 2021). Second, there is evidence that multiple-locus approaches can automatically account for cryptic relatedness without explicitly modelling the relatedness using random effect or PCs (Sillanpää 2011). Third, since multiple-locus methods can simultaneously estimate the effects of all SNPs, it is possible to derive other interesting quantitative genetic parameters such as genomic estimated breeding values (GEBVs) (Crossa et al. 2017) and genomic heritability (de los Campos et al. 2015). In fact, several multiple-locus approaches proposed for genomic prediction such as Bayes B (Meuwissen et al. 2001) and Bayes C (Habier et al. 2011) can also be used for genome-wide association analysis, because they can estimate the selection probabilities of each individual SNP to measure the degree of association between SNPs and phenotypes (Pérez and de los Campos 2014).

Linkage disequilibrium (LD) or the correlation among high-density SNPs often creates challenges for a multiple-locus approach. When the level of LD becomes high, many multi-locus methods such as penalised regression (Liu et al. 2013a) and Bayesian regression methods (Pasanen et al. 2015) become inefficient in terms of reduced power to detect QTL. To overcome this, Li et al. (2018) developed a LD-based GWAS and/or QTL mapping procedure that combines the LD analysis with either a single- or multiple-locus approach. This uses a LD network approach to firstly cluster SNPs into LD blocks and then consider the LD blocks as a unit instead of individual SNPs in the QTL analysis. The LD-based mapping approach showed better statistical power for QTL identification compared to conventional SNP-based QTL methods. In our work, we propose a new LD-based Bayesian regression method for multiple purposes including QTL identification, estimation of genomic heritability and genomic prediction. The proposed Bayesian approach is able to analyse multiple environment data and provide estimates of both main genetic effects and genotype × environment interaction (*G* × *E*) effects.

The Australian cotton industry has a reputation for growing the highest yielding cotton in the world. Continuous yield progress is attributed to genetics and crop management and by exploring their synergy (Liu et al. 2013b). Higher LP is a key factor behind the much higher yields of the most recently adopted cultivars in Australia (Conaty and Constable 2020). However, selection for higher LP leads to reduced seed weight (i.e. low seed index) (Zeng et al. 2015; Maeda et al. 2023). Seed oil and protein are two reserves for germination and seedling emergence after planting. Low seed weight therefore means reduced energy and structural building materials available in mature seeds for early plant growth, inevitably leading to poor or variable plant stand under field conditions, particularly in seasons with low or suboptimal heat conditions. This becomes a constraint for crop productivity or requires replanting of fields, at considerable cost to the grower. Such risks are expected to increase when reduced seed weight is attributed to reduced kernel weight (low seed density) or reduced seed oil content or both (Snider et al. 2014). Low seed weight and oil content has also become a major concern for post-harvest processors as low seed output after ginning and reduced oil extraction are also often associated with extra cleaning of the lint required to eliminate seed hull fragment contamination of the ginned fibre (Dowd et al. 2018; Maeda et al. 2023).

Given the role and interplay of LP, SI, LI and SOC on cotton lint, seed and oil yield and seed planting quality, in this study, we investigated their phenotypic variability and underlying genetic architecture in a MAGIC RIL population derived from four highly diverse CSIRO bred commercial varieties. A few stable and major QTL regions associated with different traits were detected; however, instead of further molecular dissection to identify specific genes or alleles associated with those QTL regions, we extended the QTL mapping to genomic prediction modelling to predict genetic worthiness of individual RILs and then the predicted breeding values were used in sequential selection with the aim of maximising lint yield while improving or maintaining seed yield and oil traits (i.e. SI and SOC). This approach was compared with the more traditional selection based on phenotypic values to identify the key genetic constraints for simultaneously improving lint, seed and oil yields in cotton. We further discuss future breeding initiatives to overcome these constraints in cotton.

## Materials and methods

### A multi-parent advanced generation intercross population and field experiments

All test lines used in this study are recombinant inbred lines (RILs) derived from a multi-parent advanced generation intercross (MAGIC) involving four released Australian cotton cultivars, namely, Sicot 71, Sicot 75, Sicot F-1 and Siokra 24. Their release year and phenotypic difference of LP, LI, SI and SOC are given in Table S1 of Supplementary file 1. The parents were initially paired to make two crosses in 2011, and resultant F_1_’s were crossed with each other to obtain 1000 hybrid seeds to form an initial population. Individuals were advanced through selfing using the single-seed descent (SSD) method to make them genetically homozygous up to *F*_6_ from which individual RILs were derived. Between the *F*_7_ and *F*_9_ generation, a subset of 256 RILs were randomly selected and tested in field experiments.

Field experiments were conducted over the summer of 2016/17, 2017/18 and 2018/19 at the Australian Cotton Research Institute near Narrabri, NSW, Australia (ACRI, S30° 11’, E149° 35’). The soil at that site is a self-mulching Vertosol classified as a fine, thermic, montmorillonitic Typic Haplustert with high clay content (Soil Survey Staff 1996). All RILs and the four parents were tested in an experiment each season with two replications according to the unresolved row–column design generated by DIGGER software. The experiment dimension is 56 rows × 10 columns, 46 × 12 and 40 × 14 in 2016/17, 2017/18 and 2018/19, respectively. Within the experiment, single row plot of 8–12 m length was used as the unit and separated by a row spacing of 1 m.

The experiments were chemically defoliated at the end of the season with thidazuron and ethephon when at least 60% of bolls were open. Hand-picking was conducted to harvest 30 fully developed, normal open bolls from each plot, and seed cotton samples were ginned with a 20-saw gin to separate lint and fuzzy seeds. The resultant lint fraction was weighed to calculate lint percentage (LP) and fuzzy seeds were acid-delinted and dried at 60 °C to reduce seed moisture before weighing. Black seeds were cleaned, and 200 undamaged mature seeds were taken to record seed index (SI), i.e. weight of 100 black seeds. Seed index and black seed weight were used to estimate total number of seeds in the samples and then in conjunction with sample lint weight to estimate lint weight per 100 seeds, i.e. lint index (LI). Finally, a subsample of black seeds with at least 15 g was taken from each plot for seed oil testing, which was done using Low-Field Time-Domain Nuclear Magnetic Resonance (Horn et al. 2011).

### Phenotype data analysis and heritability estimation

The datasets from the three-season experiments were combined together as multi-environmental trials for a pooled analysis using linear mixed model (Smith et al. 2005) as follows:

1 $$y=X\tau +Z\mu +e$$

where $\tau =({\tau}_{\eta}^{\prime}, {\tau}_{p}^{\prime}{)}^{\prime}$ represents the vector of fixed effects of season (or experiment,${\tau }_{\eta }$) and experiment-specific peripheral effects (${\tau }_{p})$) associated with design matrix *X* = ${[X}_{\eta },{X}_{p}]$, and $\mu =({\mu }_{\eta}^{\prime},{\mu }_{p}^{\prime}{)}^{\prime}$ represents the vector of random effects of test line in the experiment $({\mu }_{\eta })$ and experiment-specific non-genetic effects (or peripheral, ${\mu }_{p}$) associated with design matrix *Z* = $[{Z}_{\eta }{,Z}_{p}]$ and *e* is the combined vector of plot error effects from each experiment. In the analysis, season was treated as a fixed effect and test line and their interactions were treated as random effects. Usually, ${\tau }_{p}$ is to model any large trend existing along the row and column of the experiment, by adding covariates in the part of fixed terms of the model; ${\mu }_{p}$ is to model the random effect from replicate, row and column so as to count for replication and inter-block effect. The analysis assumed the correlation model of an autoregressive process of order one for row and column indexed experiment plots to take account of spatial variation and heterogeneity of residual error variances of different experiments (see the full detail in Smith et al. (2005) and Liu et al. (2015c)). When the best models were resolved by log-likelihood ratio test, variance components for genotype and its interaction with test season were obtained for heritability estimation as follows. Meanwhile, empirical best linear unbiased estimates or adjusted means for test lines (256 RILs and four parents) were obtained for individual and pooled experiments, after switching genotype and its interaction terms in the model as fixed. These means were then considered as phenotype values in the subsequent QTL and GP analyses.

Narrow sense heritability (*h*_N_^2^) and genomic heritability (*h*_g_^2^) were estimated in this study. *h*_N_^2^ represents the ratio of *σ*^2^_additive_/*σ*^2^_p_, where *σ*^2^_additive_ and *σ*^2^_p_ are additive and phenotypic variance estimate, respectively. The *σ*^2^_g_ was available in the output of the combined analysis as aforementioned. The *σ*^2^_additive_ was obtained when considering the approximation of *σ*^2^_additive_ to half of *σ*^2^_g_ in a RIL population derived from a bi-parental cross (Bernardo 2002) to remain true for a MAGIC RIL population in this study, under the no epistasis assumption. The *σ*^2^_p_ was the sum of ${\sigma }_{g}^{2}+\frac{1}{2}\widetilde{\upsilon }$, where $\widetilde{\upsilon }$ represents the mean variance of a difference between a pair of adjusted means, which can in nature reflect and account for heterogeneity of the residual error variance of different experiments as well as spatial variations fitted in a model for the combined data (Cullis et al. 2006; Piepho and Mӧhring 2007). The $\widetilde{\upsilon }$ was obtained from averaging standard error of a difference for all pairwise adjusted means (s.e.d) available in ASReml prediction output for the term of genotype. The standard error for *h*_N_^2^ was obtained based on the Delta method (Holland 2003). *h*_g_^2^ was estimated by using the Average Semivariance (ASV) estimator (Feldmann et al. 2022) described in the section “Bayesian multiple-locus single environmental model”. All the analyses were carried out using ASreml-R (Butler et al. 2009) and our in-house R code, respectively.

### Genotyping

Leaf samples of the 256 RILs (F_9_ generation) were collected in the 2018/19 season. After freeze-drying, the leaf samples were supplied to Diversity Arrays Pty Ltd. (DArT, Canberra, Australia) for DNA extraction and genotyping using the company’s proprietary methods and protocols. The genotyping was done using DArTagtm, a genotyping-by-sequencing platform designed based on a custom array of 8873 single nucleotide polymorphism (SNP) markers selected based on whole genome resequencing data of a set of diverse Australian cotton varieties and breeding lines, many of which were previously incorporated in the publicly available CottonSNP63K array (Hulse-Kemp et al. 2015). SNP calling was performed according to the DArT standard protocols. SNPs with a missing genotype data rate of more than 20% and a minor allele frequency less than 2.5% were filtered out, and the rest of the missing genotype data were imputed using the software Beagle, version 5.4 (Browning et al. 2018; http://faculty.washington.edu/browning/beagle/beagle.html).

### SNP clustering by linkage disequilibrium network

In large genomic datasets, physically adjacent SNPs are often in linkage disequilibrium (LD). Since a group of SNPs in high LD explains similar amounts of genetic variation in a given trait and likely corresponds to a single functional unit, it is reasonable to account for such correlation structure among the SNP data in QTL analysis. We used LD network clustering (LDn-clustering) as a tool to derive LD blocks among SNPs, which were incorporated into the QTL mapping (Li et al. 2018). Briefly, the LDn-clustering approach started by dividing each chromosome into roughly equal-sized and non-overlapped windows. Within each window, the pairwise LD measure in terms of the *r*^2^ parameter among SNPs was calculated. Based on the pairwise LD, the blocks consisting of SNPs with high LD were identified. These LD blocks were then used as basic units in the Bayesian multiple-locus model described below. In practice, the method was implemented using the function “LDnClustering” in the R package LDna (Kemppainen et al. 2015; https://github.com/petrikemppainen/LDna). In the function LDnClustering, we used the default setting of the parameters proposed in the LDna package as *r*_1_ = 0.5 (minimum LD value within a cluster), *r*_2_ = 0.7 (minimum median LD within each cluster), *w*_1_ = 10 (window size for defining putative recombination hotspots), and *w*_2_ = 100 (window size for estimating LD values).

### Bayesian multiple-locus multiple environmental model

We propose a new LD-Bayes method (Bayesian LD-based multiple-locus linear mixed model) to analyse multiple environment QTL data:

2 $${y}_{ij}={\beta }_{0}+{\beta }_{0j}+{\alpha }_{ij}+\sum\limits_{k=1}^{p}\sum\limits_{l=1}^{{q}_{k}}{x}_{ikl}{\beta }_{kl}+\sum\limits_{j=1}^{m}\sum\limits_{k=1}^{p}\sum\limits_{l=1}^{{q}_{k}}{x}_{ijkl}{\beta }_{jkl}+{e}_{ij}$$

where *y*_*ij*_ is the phenotype of line *i* (*i* = 1,…, *n*_*j*_) at the environment *j* (*j* = 1, 2,…, *m*), and *x*_*ijkl*_ (= *x*_*ikl*_) is the genotype value of SNP *l* (*l* = 1,…, *q*_*k*_) located within the LD block *k* (*k* = 1,…, *p*) of the line *i*, coded as *x*_*ijlk*_ = − 1, 0, 1 for genotypes AA, AB and BB, respectively. *β*_0_ is the fixed intercept term representing the population mean. *β*_0*j*_ is the environment specific population mean. ${\alpha }_{ij}$ is the random intercept term specified for each individual line *i*, ${\beta }_{kl}$ is the main additive genetic effect of SNP *l* in the LD block *k*, *β*_*jkl*_ is genotype by environmental interaction effect, i.e. the genetic effect of SNP *l* in the LD block *k* specific at the environment *j*, and *e*_*ij*_ is the residual which is mutually independent and follows a normal distribution N(0,${\sigma }_{0}^{2}$), with unknown variance ${\sigma }_{0}^{2}$.

Model (2) can be specified as a likelihood function:

3 $$P\left(y|{\beta }_{0},{\alpha }_{ij},{\beta }_{jkl},{\sigma }_{0}^{2}\right)=\prod\limits_{{i = 1}}^{n}\frac{1}{\sqrt{2\pi {\sigma }_{0}^{2}}}{\text{exp}}\left(\frac{-{\left({y}_{i}-{\beta }_{0}-{\beta }_{0j}-{\alpha }_{ij}-{\sum }_{k=1}^{p}{\sum }_{l=1}^{{q}_{k}}{x}_{ikl}{\beta }_{kl}-{\sum }_{j=1}^{m}{\sum }_{k=1}^{p}{\sum }_{l=1}^{{q}_{k}}{x}_{ijkl}{\beta }_{jkl}\right)}^{2}}{2{\sigma }_{0}^{2}}\right)$$

In Bayesian statistics, all the model parameters were assigned with prior distributions, and the priors are combined with the likelihood to form the posterior distribution as

4 $$P\left({\beta }_{0},\alpha ,\beta ,{\sigma }_{0}^{2}|y\right)\propto P\left(y|{\beta }_{0},\alpha ,\beta ,{\sigma }_{0}^{2}\right)p\left({\beta }_{0}\right)p\left(\alpha \right)p\left(\beta \right)p\left({\sigma }_{0}^{2}\right).$$

The intercept terms ${\beta }_{0j}$ were assigned with a non-informative uniform prior as ${\beta }_{0j}\sim Uni\left(-\infty ,\infty \right)$.

The additive genetic effect ${\beta }_{jkl}$ (as well as ${\beta }_{kl}$) was assigned with a spike and slab prior (Ishwaran and Rao 2005; O’Hara and Sillanpää 2009), a mixture distribution of a normal distribution and point mass at zero as:

5 $$P\left({\beta }_{jkl}|{\gamma }_{jkl}\right)\propto {\gamma }_{jkl}N\left({\beta }_{jkl}|0,{\sigma }_{jk}^{2}\right)+\left(1-{\gamma }_{jkl}\right){I}_{\left({\beta }_{jkl}=0\right)},$$

where γ_*jkl*_ (= 0 or 1) is a binary indicator variable. If* γ*_*jkl*_ = 1, the SNP effect ${\beta }_{jkl}$ is supposed to be non-trivial and follows a normal distribution $N\left({\beta }_{jkl}|0,{\sigma }_{jk}^{2}\right)$, and the effect of correlated SNPs in the same LD block *k* (at a given environment *j*) was assumed to have the same variance ${\sigma }_{jk}^{2}$. If γ_*jkl*_ = 0, the effect becomes zero and is excluded from the model.

In (5), the indicator variable γ_*jkl*_ was further assigned with a Bernoulli prior:

6 $$P\left({\gamma }_{jkl}\right)={\omega }^{{\gamma }_{jkl}}{\left(1-\omega \right)}^{1-{\gamma }_{jkl}},$$

where the parameter $\omega$ can be interpreted as the proportion of the regression parameters to be assigned with non-zero effect. It was assigned with a hyperprior a Beta distribution:

7 $$P\left(\omega \right)=Beta\left(\omega |a,b\right),$$

with *a* = *b* = 25. The mean of the prior is *a*/(*a* + *b*) = 0.5, so there is no preference whether a SNP should be selected into the model.

The individual specific random intercept effect ${\alpha }_{ij}$ was assumed to independently follow a normal distribution as

8 $${\alpha }_{ij}\sim N\left(0,{\sigma }_{\alpha }^{2}\right).$$

This setting introduces a homogeneous covariance structure among the phenotypes over different environments as COV(*y*_*ij*_, y_*ij*_) = ${\sigma }_{0}^{2}+{\sigma }_{\alpha }^{2}$, and COV(*y*_*ij*_, *y*_*ik*_) = ${\sigma }_{0}^{2}$.

The variance components ${\sigma }_{0}^{2}$, ${\sigma }_{jk}^{2}$ and ${\sigma }_{\alpha }^{2}$ in (3), (5) and (8) are all assigned with Scaled inverse chi-squared prior:

9 $$P\left({\sigma }^{2}\right)=Inv-{X}^{2}({\sigma }^{2}|c,d)$$

with rate parameter c fixed to be 5/2 for all the three variant components. The scale parameter was specified as ${d}_{0}=\text{var(y)}\times \left(1-{R}^{2}\right)\times \left( \, {\text{c}}+1\right)$, ${d}_{\alpha }=\frac{5}{2}$ and ${d}_{jk}=\text{var(y)}\times {R}^{2}\times \left( \, {\text{c}}+1\right)$/mean(diag(G)), where *G* = *X*^T^*X*, and *X* is the SNP matrix with rows corresponding to individuals and columns corresponding to SNPs. *R*^2^ is model assumption of the proportion of phenotype variance explained by the genetic component.

These hyperparameter settings were suggested in Pérez and de los Campos (2014) as default for Bayes B or C models for genomic prediction.

Since all the priors used were conjugate, the posterior can be evaluated using a Markov Chain Monte Carlo (MCMC), or more specifically a Gibbs sampling algorithm, which is described in Supplementary file 2. In practice, we simulated 60,000 samples, with the first 10,000 samples considered as burn-in, and the remaining 50,000 were thinned at every 50th sample to reduce the serial correlation. Consequently, 1000 samples were finally obtained for the posterior inference.

### QTL decision rules

From the LD-Bayes model (4), the MCMC algorithm outputted dependent samples of model parameters representing their posterior distribution. The posterior mean of the indicator variable ${\gamma }_{jkl}$ can usually be interpreted as a posterior inclusion probability (Guan and Stephens 2011) of the regression parameter ${\beta }_{jkl}$ (the additive genetic effect of marker *l* in the LD block *k* at the environment *j* to be presented in the model). Since the SNPs in each LD block are highly correlated with each other, it is reasonable to assume those SNPs represent the same QTL. To conduct the QTL judgement at the LD block level, we calculated the probability of at least one SNP from a LD block was included in the model:

10 $$P\left({Z}_{jk}=1|Y\right)=P\left(\cup {\gamma }_{jkl}=1;\quad l=1,\ldots ,{q}_{k}\right)$$

where *Z*_*jk*_ is a binary indicator variable to tell whether the LD block *k* should be included in the model or not. Note that here we focus only on detecting significant genomic regions from each separate environment, so the label *j* was considered as fixed in the rest of this session. Another quantity for model selection named Bayes factor (BF) (Kass and Raftery 1995) can be defined as

11 $$B{F}_{jk}=\frac{P\left(Y|{Z}_{jk}=1\right)}{P\left(Y|{Z}_{jk}=0\right)}=\frac{P\left({Z}_{jk}=1|Y\right)P\left({Z}_{jk}=0\right)}{P\left({Z}_{jk}=0|Y\right)P\left({Z}_{jk}=1\right)}$$

where $P\left({Z}_{jk}=0\right)={\left(1-\widehat{\upomega }\right)}^{{q}_{k}}$, and *P*(*Z*_*jk*_ = 1) = 1−*P*(*Z*_*jk*_) = 1−(1−$\widehat{\upomega }$)^*qk*^, $\widehat{\upomega }$ is the posterior mean estimate of *ω*, defined in Eq. (6). The BF_*jk*_ was often interpreted at the log_10_ scale. If log_10_(BF_*jk*_) > 0 (or BF_*jk*_ > 1), it indicates that *Z*_*jk*_ = 1 is in favour of *Z*_*jk*_ = 0. When log_10_(BF_*jk*_) > 2 (or alternatively saying BF_*jk*_ > 100), it is believed that the conclusion of *Z*_*jk*_ = 1 in favour of *Z*_*jk*_ = 0 is decisive (Kass and Raftery 1995). Hence, the log_10_(BF_*jk*_) > 2 can be used as a criterion to declare a QTL. This simple decision rule might be limited because it does not account for the multiplicity, i.e. the fact that thousands of decisions (i.e. the total number of LD blocks *p*) were conducted simultaneously may result in the high chance of false positive detections by random chance (Scott and Berger 2010). To overcome this, a Bayesian false discovery rate (FDR) control approach (Ventrucci et al. 2011; Wen 2017; Chen et al. 2021) was also applied here to account for the multiplicity.

A multiple hypothesis testing problem is defined as

Null: ${Z}_{jk}=0$ vs Alternative: ${Z}_{jk}=1$ for *k* = 1, …, *p*

The probability $P\left({Z}_{jk}=0|Y\right)=1-P\left({Z}_{jk}=1|Y\right)$ can be interpreted as a local FDR specified for the genomic region *k*. The Bayesian FDR is then defined as

12 $$BFDR=\frac{{\sum }_{k=1}^{p}{\delta }_{k}\left(t\right)P\left({Z}_{jk}=0|Y\right)}{{\sum }_{k=1}^{p}{\delta }_{k}\left(t\right)}$$

where, ${\delta }_{k}\left(t\right)={I}_{\left(P\left({Z}_{jk}=0|Y\right)<t\right)}$ is a binary decision rule which controls the BFDR at the level α (e.g. *α* = 0.05) with the threshold

13 $${t}_{\alpha }=\underset{t}{min}\left({\text{BFDR}}<\alpha \right)$$

According to (12) and (13), the following procedure was used for the BFDR control:

(i) The Local FDR $P\left({Z}_{jk}=0|Y\right)$ was sorted in ascending order as ${\text{LFDR}}_{\left(1\right)},\dots , {\text{LFDR}}\left(m\right)$.
(ii) The first *r* genomic regions were declared as QTL which satisfied
  $$\frac{{\sum }_{j=1}^{r}{{\text{LFDR}}}_{\left(j\right)}}{r}<\alpha .$$

A genomic region was declared as a major QTL region when the above BFDR procedure has a *α* ≤ 0.05.

### Genomic heritability estimation

The genomic heritability was estimated using the ASV estimator introduced in Feldmann et al. (2022):

14 $${{h}_{g}}^{2}=\frac{{\left(n-1\right)}^{-1}tr\left(G\right)\widehat{{\sigma }_{g}^{2}} }{{\left(n-1\right)}^{-1}tr\left(G\right)\widehat{{\sigma }_{g}^{2}} +\widehat{{\sigma }_{e}^{2}}},$$

where *n* is the number of lines used in the analysis, *G* is the genomic relationship matrix (VanRaden 2008), and $\widehat{{\sigma }_{g}^{2}}$ and $\widehat{{\sigma }_{e}^{2}}$ are the posterior estimates of genetic variance and residual variance using Bayesian genomic linear unbiased prediction (Pérez and de los Campos 2014) with the response variables as the average of three-year phenotype data. Since this generates an empirical distribution of *h*_g_^2^, we considered the mean of *h*_g_^2^ as the point estimates of genomic prediction. The standard error can also be easily calculated.

### Genomic prediction

The LD-Bayes model (4) can also be used for genomic prediction. In fact, it is similar to the Bayes C model in the GP literature (Pérez and de los Campos 2014), but with the difference that the LD information is taken into consideration and SNP effects within each LD block are assigned with separate variance components. In contrast, in Bayes C, all the SNP effects are assumed to follow a normal distribution with a common variance.

To evaluate the predictive ability of the models, we considered the following two scenarios. The first scenario is a fivefold cross-validation (CV) procedure to randomly divide the population into five parts with roughly equivalent size. In turn, each part was considered as test population, and the rest were considered as the training population with phenotype data in the 2016/17 and 2017/18 seasons as known. The prediction accuracy was defined as the Pearson correlation between the GEBVs and the real phenotype data at 2018/19 season in the test population divided by the square root of *h*_g_^2^.

In the second scenario, in turn, we considered all the genotype data and single, two and three seasons’ phenotype data as the training population to evaluate GEBVs. The GEBVs were then used to select best performed lines with the improved combinations of the target traits, and results were compared to the selection based on adjusted phenotypic means.

### Phenotypic correlation and network analysis

Trait interrelation can affect simultaneous improvement of their combinations of interest in this study, Pearson’s correlation analysis was conducted using phenotypes of single and across season experiments to examine the nature and magnitude of trait interrelations as well as how they were affected by test season. The directed phenotype network was constructed using the well-known PC algorithm for causal inference (Kalisch and Buehlmann 2007) based on phenotypic means of a pooled analysis as well as genomic regions for QTLs with proportion of phenotype variance explained (PVE) larger than 0.04 for each trait. The algorithm was implemented by the function pc() in the R package ‘pcalg’ (Kalisch et al. 2012), with the significance threshold specified as *α* = 0.01 in the conditional independence tests.

### Simulation studies for QTL mapping and genomic prediction

A simulation study was used to evaluate the performance of LD-Bayes in terms of both QTL mapping and GP and to compare its performance to common Bayesian regression approaches proposed in quantitative genetics. Two-season phenotype data were simulated on the basis of the genotype data of our MAGIC population. In total, additive genetic effects were simulated at 55 SNPs, located in 10 different LD blocks (Table S2). In brief, 5 SNPs located in 5 different LD blocks including 3 common QTLs with effects on the phenotypes in both seasons, and 2 *G* × *E* QTLs with season specific effects were assigned with major genetic effects, simulated from a normal distribution *N* (5, 2.25) (Table S2). Additionally, we also simulated minor genetic effects from a normal distribution *N* (1.5, 0.25) for additional 50 SNPs, with every 10 SNPs distributed in 5 separate LD blocks. 30 of these SNPs have a common genetic effect across two seasons, and the other 20 have *G* × *E* effects. We further simulated a residual error term from a normal distribution *N* (0, 100), independently per season and per individual. In regard to the QTL analysis, the performance of LD-Bayes was compared to Bayes B (Meuwissen et al. 2001) and Bayes C (Habier et al. 2011), while for genomic prediction, LD-Bayes was further compared to the Bayesian Genomic Best Linear Unbiased Prediction (BG-BLUP) (Pérez and de los Campos 2014) in addition to Bayes B and Bayes C. Similar to LD-Bayes and Bayes C, Bayes B used a spike and slab prior on each SNP effect, as a mixture of point mass and zero and a student t distribution, while BG-BLUP is on the basis of a random effect model, by linking the genomic relationship matrix calculated using the genome-wide SNP data to the phenotypes, instead of estimating the effects of each individual SNP.

In QTL mapping, the LD block level BF is calculated according to Eq. (11) for LD-Bayes, while for Bayes B and Bayes C, BF was calculated in the individual SNP level. A SNP was declared as significant when BF > 100 or alternatively saying when log_10_(BF) > 2, the same criterion was used to declare a suggestive QTL in the real data analyses. If the significant SNP was located in the same LD block as a true simulated QTL, we claimed that the QTL was correctly identified by a method. If the significant SNP did not belong to any LD block where a true QTL was simulated, it was considered as a false positive. In genomic prediction, the same cross-validation strategy as used in the real data analyses was used here to evaluate the prediction accuracies.

The whole simulation process was repeated for 50 times, and the average performance of the LD-Bayes method was summarised to evaluate whether the method has the power to identify each QTL region, and control the false positives, as well as its ability for genomic prediction.

## Results

### Phenotypic variation and heritability of yield components and seed oil content traits

Large phenotypic difference existed in the population for all traits (Table 1). Variability of LP, SI and SOC significantly deviated from a standard normal distribution due to strong skewness and kurtosis, but with the population means close to those of Sicot 75, a parent having the highest LP and lowest SI and SOC (Table S1). The variation of LI followed a standard normal distribution with mean approximate to that of parents (7.6 vs 7.7 g lint/100 seeds).

**Table 1:** Mean, variability and heritability of three seed yield traits and oil content in a MAGIC population of cotton

Trait	Mean	Range	Number of lines		Skewness	Kurtosis	*h*_N_^2^	*h*_g_^2^
			≤ Low parent	≥ High parent				
Lint percentage (%)	42.4	32.7–47.0	2 (0.8)	131 (51.2)	− 0.91***	8.26***	0.472 ± 0.004	0.443 ± 0.033
Seed index (g/100)	8.5	7.1–11.1	108 (42.2)	2 (0.8)	0.78***	5.64***	0.470 ± 0.004	0.483 ± 0.037
Lint index (g/100)	7.6	6.4–9.0	6 (2.3)	6 (2.3)	0.16^ ns^	2.82^ ns^	0.462 ± 0.005	0.481 ± 0.037
Seed oil content (%)	19.7	17.6–25.1	131 (51.2)	0 (0.0)	0.87***	6.40***	0.455 ± 0.005	0.380 ± 0.029

^ns^means non-significant; ***represents *P* < 0.001

Proportionally, there are 51% more in the population with LP higher than the highest parent, Sicot 75, while 42% and 51% of individuals had lower SI and SOC than the lowest parent, Sicot 75. Therefore, abundant RIL lines with high LP but low SI and SOC existed in the population. Overall, only 2.3% of the population exhibited better or worse than the highest or lowest parent, respectively. Narrow sense and genomic heritability estimates (*h*_N_^2^ and *h*_g_^2^) stayed in moderate range and comparable for SI and LI but higher *h*_N_^2^ for LP and SOC (Table 1).

### Genotyping and linkage disequilibrium analysis

The DArT genotyping array contains 8873 SNPs. After data filtering and pre-processing, 6523 informative SNPs distributed over 26 chromosomes of *G. hirsutum* were kept and used in the subsequent analysis. The LD network analysis classified the SNPs into 2048 clusters, with 47 to 119 clusters on different chromosomes (Table S3). The clusters contain unequal numbers of SNPs, with 8 clusters each comprising over 50 SNPs and 805 clusters only represented by a single SNP. The clusters or LD blocks were applied to the QTL mapping and genomic prediction.

### QTL mapping

**Fig. 1:**
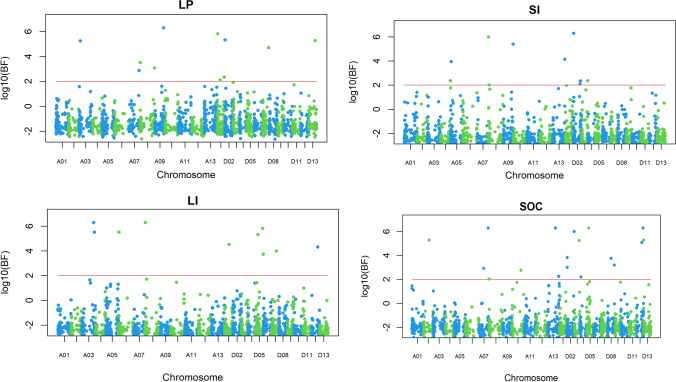
QTLs and their genomic regions for lint percentage (LP), seed index (SI), lint index (LI) and seed oil content (SOC) using the Bayesian multiple-locus multiple environment model. *BF* Bayes factor

QTL analyses were done by applying the multiple environment Bayesian model proposed in this study to the phenotypic dataset consisting of estimated means of three-season experiments. For all four traits, no significant genotype × environment interaction effects were detected. Using the BFDR approach, overall, 7, 7, 8 and 10 genomic regions or common QTLs were identified to be associated with LP, SI, LI and SOC, respectively (Table 2; Fig. 1). The number of QTLs identified in At and Dt subgenomes was similar for LP and SI but higher in Dt for LI and SOC. Each of these QTLs explained 1–12% of PVE, with 3 (LD-223, LD-1071 and LD-2032), 2 (LD-723 and LD-1172), 2 (LD-245 and LD-252) and 4 (LD-157, LD-554, LD-1178 and LD-1661) QTLs having a PVE ≥ 5% for LP, SI, LI and SOC, respectively, and they were considered as major QTLs contributing to the corresponding trait.

**Table 2:** QTLs and their genomic regions for lint percentage, seed index, lint index and seed oil content

Trait	Type of effect	Genomic region ID	Chromosome (Positions in kb)	Log_10_ (Bayes factor)	PVE^a^
Lint percentage	Main effect	223	A03 (9371–13,325)	5.24	**0.08**
		554	A07 (82,211)	2.87	0.01
		632	A08 (118,937–119,062)	3.08	0.02
		1071	D01 (6894–6928)	5.82	**0.11**
		1157	D02 (2844–3139)	5.34	0.04
		1609	D07 (48,132–52,450)	4.70	0.03
		2032	D13 (51,833–53,536)	5.29	**0.05**
Seed index	Main effect	357	A05 (5187–5250)	3.95	0.04
		559	A08 (436–481)	6.00	0.04
		723	A09 (80,953–81,145)	5.39	**0.05**
		1037	A13 (100,095)	4.15	0.04
		1102	D01 (32,638–43,811)	0.80	0.02
		1172	D02 (5250)	6.30	**0.06**
		1225	D02 (62,001–62,890)	2.35	0.04
Lint index	Main effect	245	A03 (93,792)	6.30	**0.07**
		252	A03 (100,032–100,493)	5.52	**0.05**
		467	A06 (2839–3969)	5.15	0.03
		1106	D01 (35,829)	4.52	0.02
		1433	D05 (20,767–21,050)	5.34	0.04
		1486	D05 (64,033–64,088)	3.73	0.04
		1606	D07 (29,364–45,029)	3.98	0.03
		1915	D12 (46,158–48,682)	4.31	0.03
Seed oil content	Main effect	157	A02 (47,738–67,435)	5.29	**0.05**
		554	A07 (82,211)	6.30	**0.12**
		1001	A13 (68,680)	6.30	0.03
		1178	D02 (6764–7124)	3.83	**0.08**
		1261	D02 (7008–70,089)	5.99	0.03
		1296	D03 (41,854–43,670)	5.25	0.03
		1426	D05 (19,058)	6.30	0.04
		1661	D08 (25,811–51,164)	3.76	**0.06**
		1915	D12 (46,158–48,682)	5.08	0.03
		1980	D13 (439–1128)	5.30	0.02

^a^Proportion of phenotype variance explained by a QTL region with those ≥ 0.05 shown in bold font

Overlapping or adjacent genomic regions contributing to two traits were found in seven chromosomes (Table 2). For examples, LP and SOC shared LD-554 on A07 and LI and SOC shared LD-1915 on D12, and on D01, an LI QTL (LD-1106) overlapped with an SI QTL (LD-1102); two adjacent regions were mapped for SI and SOC on D02 (LD-1172 and LD-1178, LD-1225 and LD-1261), and one on D05 (LD-1426 and LD-1433); on D07 an LI QTL (LD-1606) was next to an LP QTL (LD-1609).

### Phenotypic interrelation and network

To simultaneously improve lint, seed and oil yields in cotton, breeders require to watch how selection for one trait can affect the other. Therefore, it is important to understand the nature and magnitude of interrelations of breeding target traits. In this study, three pairs of traits, LP vs SI, LP vs SOC and LI vs SOC, were inversely related with the magnitude from moderate to weak in the listed order (Table 3). The other three trait pairs showed positive relations but to a moderate extent for LP vs LI and SI vs LI and to a weak extent for SI and SOC. Interestingly, the nature of the above trait relations did not alter with the season of the experiments.

**Table 3:** Correlation coefficients between three yield traits and seed oil content of individual and pooled season experiments

Trait/season	Seed index	Lint index	Seed oil content
*Lint percentage*			
2016/17	− 0.509***	0.494***	− 0.316***
2017/18	− 0.481***	0.377***	− 0.347***
2018/19	− 0.581***	0.398***	− 0.348***
Overall	− 0.560***	0.465***	− 0.393***
*Seed index*			
2016/17		0.384***	0.251***
2017/18		0.389***	0.233***
2018/19		0.329***	0.239***
Overall		0.367***	0.282***
*Lint index*			
2016/17			− 0.133*
2017/18			− 0.085
2018/19			− 0.215***
Overall			− 0.174**

*, **, *** represent *P* < 0.05, 0.01 and 0.001, respectively

**Fig. 2:**
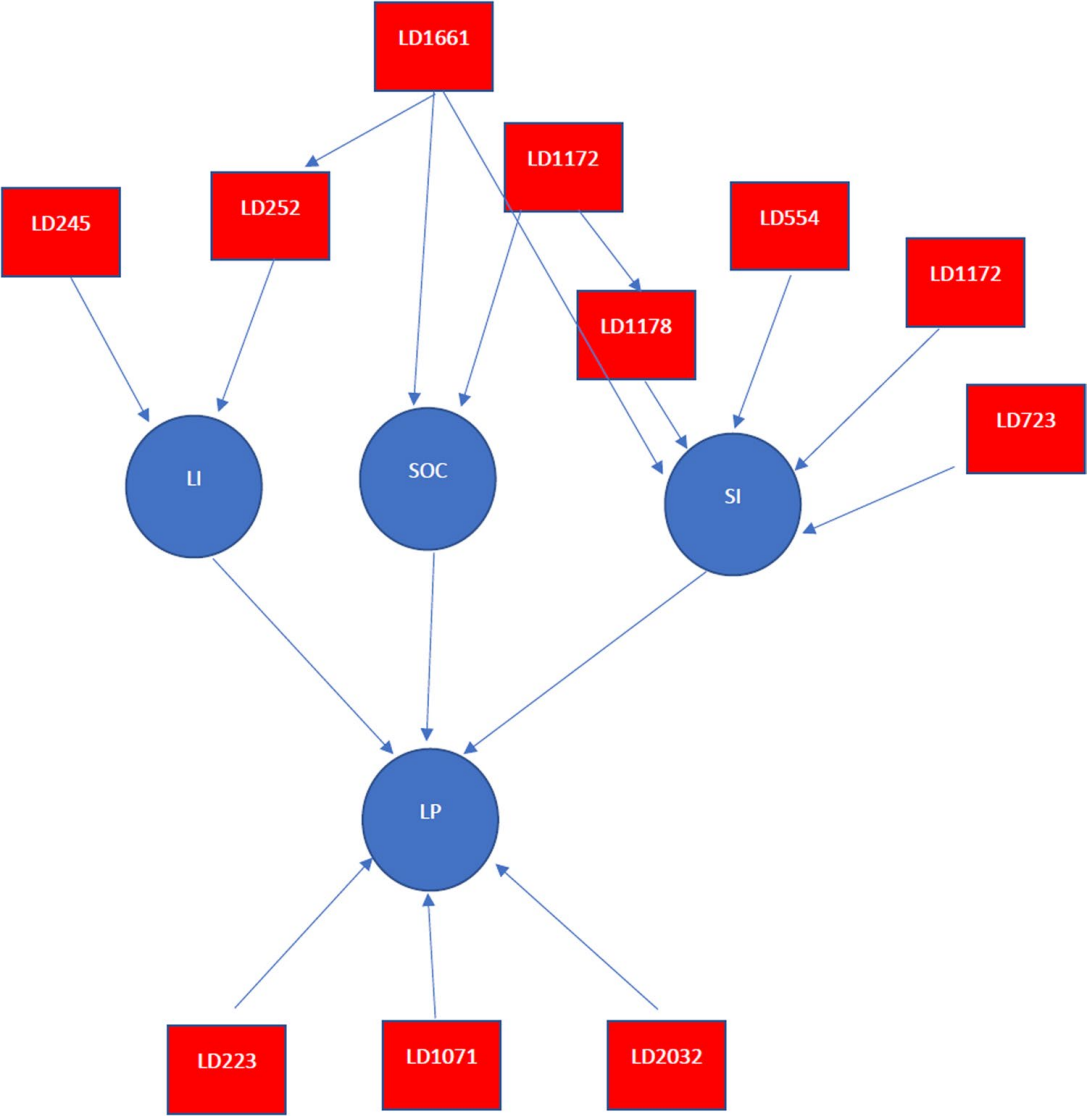
Phenotype network constructed by causality inference. Blue circles represent traits including lint percentage (LP), lint index (LI), seed oil content (SOC) and seed index (SI), and red rectangles represent LD blocks (QTL regions) linked to each trait

The influence of major QTLs on the traits can be either direct or indirect (Fig. 2). For LP, three QTLs on chromosome A03 (LD-223), D01(LD-1071) and D13 (LD-2032), respectively, showed direct influence; however, the one on chromosome A07 (LD-554) exerted its influence via SI. Two QTLs (LD-723, LD-1172) for SI showed direct influence, and this was the same of two QTLs (LD-245, LD-252) for LI. For SOC, only one (LD-1661) of the three major QTLs gave direct influence, however, it also influenced SI directly and LI indirectly via one of the LI QTLs, i.e. LD-252. Furthermore, the other key SOC QTLs (LD-554, LD-1178) also directly influenced SI (Table 2; Fig. 2). This causal network confirmed known phenotypic relations between different traits (Table 3) and also suggests indirectly exploiting genetic variation associated with one trait may effectively improve the others, for example, selection for higher SOC via higher SI.

### Genomic prediction

In fivefold cross-validation (CV), the average prediction accuracies ranged from 0.46 to 0.62 across traits for the LD-Bayes method developed in this study (Table 4). Notably, the LD-Bayes approach provided 5–8% better prediction accuracies compared to the results of the classical Bayes C model.

**Table 4:** Genomic prediction results by fivefold cross-validation and the prediction accuracy

Cross validation (5 folds)	Lint percentage	Seed index	Lint index	Seed oil content
LD-Bayes	0.46	0.62	0.56	0.50
BayesC	0.43	0.59	0.52	0.47

The accuracy is defined as Pearson correlation between the GEBVs and phenotypic means of the year 2018/19 experiment divided by the square root of the genomic heritability

When phenotypic data are available, the LD-Bayes model can generate the genomic estimated breeding values (GEBVs) for test lines. In this study, we first generated GEBVs of RIL population using phenotypic means of single, paired and all three-season experiments. In breeding practice, phenotypic means of a pooled analysis is commonly used to proceed selection decision (Smith et al. 2005). To examine the usefulness of GEBVs for selection, we correlated them with phenotypic means of a pooled analysis. The correlation coefficient (*r*) ranged from 0.64 to 0.84 when GEBVs were obtained from using single-experiment phenotypic means (*df* = 254). The *r* increased to a range of 0.83–0.92 (*df* = 254), when using any two-season phenotypic means, and reached almost perfect range of 0.96–0.98, when using all three-season phenotypic means. The regression plots in Supplementary file 1 Figs. S1, S2 and S3 highlighted such improved perfectness, suggesting very high accuracy of GPs under the perfect and unrealistic scenario where training and test populations are the same. To illustrate how selection based on GEBVs worked, in the following section, we compared selections based on GEBVs as well as phenotypic means to demonstrate the accuracy for GPs required so that it became as competitive as phenotypic means when used for identifying elite RILs with improved combination for seed traits as well as lint yields in this study.

### RILs with improved LP, SI and SOC combinations retained by different selection strategies

Four selection scenarios were conducted based on either GEBVs or phenotypic means, with the key aims of capturing the individual RILs with SI ≥ 8.6 g/100 and SOC ≥ 19.0% under the selection cut points for LP of ≥ 41.0% and 42%, respectively (Table 5). The thresholds for SI and SOC were chosen, under the consensus view that their higher values are important to seed germination and early seedling vigour (Snider et al. 2016; Maeda et al. 2023). When referring to the ranges reported in commercial cultivars and cotton germplasm, a SI of 8.5 g/100 is the minimum measured with fuzzy seeds (Bourland et al. 2022; Maeda et al. 2023) and SOC of 19.1% is the minimum observed in the germplasm panel sets used for association studies (Ma et al. 2019; Zhao et al. 2019). Due to the shrinkage nature of GEBVs, selection cut points for genomic selection were determined through the linear regression equation of the phenotypic means with GEBVs (Figs. S1, S2 and S3).

**Table 5:** Number of RIL lines retained when selecting for three yield traits sequentially based on genomic predictions of 2016/17 and 2018/19 season phenotyping and phenotypic means of a pooled analysis of three-season experiments

Truncation value for sequential selection of			No lines	Reduced number of the lines by selection for		
Lint percentage (%)	Seed index (g/100 seeds)	Oil content (%)	Retained	Lint percentage	Seed index	Oil content
*Genomic predictions*^*a*^						
40.1	8.8	19.6	54 (21.1)			
40.1	8.8	20.4	24 (9.4)			− 30
40.1	9.1	19.6	15 (5.8)		− 39	
40.1	9.1	20.4	6 (2.3)		− 18	− 9
40.8	8.8	19.6	36 (14.1)	− 18		
40.8	8.8	20.4	14 (5.4)	− 10		− 22
40.8	9.1	19.6	8 (3.1)	− 7	− 26	
40.8	9.1	20.4	2 (0.8)	− 4	− 12	− 6
*Phenotypic means*						
41.0	8.6	19.0	48 (18.8)			
41.0	8.6	20.0	20 (7.8)			− 24
41.0	9.0	19.0	14 (5.5)		− 34	
41.0	9.0	20.0	6 (2.3)		− 14	− 8
42.0	8.6	19.0	34 (13.3)	− 14		
42.0	8.6	20.0	13 (5.1)	− 7		− 21
42.0	9.0	19.0	10 (3.9)	− 4	− 24	
42.0	9.0	20.0	5 (2.0)	− 1	− 7	− 5

Value in bracket represents the proportion of test lines kept from the population (%)

^a^Selection truncation points for LP, SI and SOC of genomic selection were determined by applying regression equation for predicted value and phenotypic results presented in Fig. S2

**Fig. 3:**
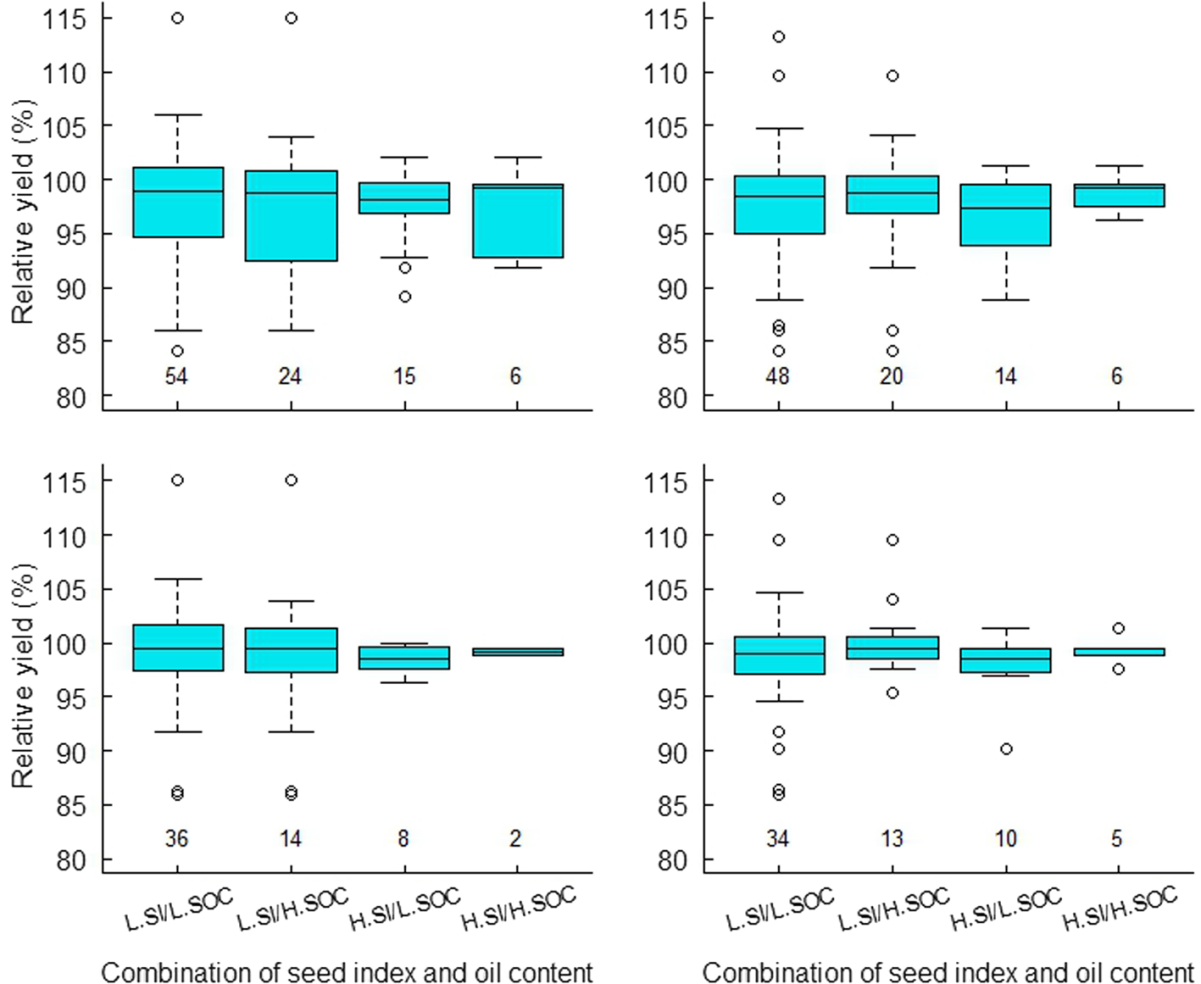
Relative lint yield variation of the retained RIL lines after simultaneous selection for lint percentage, seed index and seed oil content based on genomic prediction of the 2016/17 and 2018/19 season phenotyping (left panel) and phenotypic means of a pooled analysis of three-season experiments (right panel). Relative lint yield was calculated against Sicot 71, the highest yielding parent (2372.2 kg/ha) based on a pooled analysis. Top and bottom panels represent selection scenarios for three seed yield traits under two levels of lint percentage, while combined with low and high seed index and seed oil content. The truncation points for low and high lint percentages are 40.1% (left, top panel) and 40.8% (left, bottom panel) for genomic selection and 41% (right, top panel) and 42% (right, bottom panel) for phenotypic selection; for low and high seed indices (L.SI and H.SI) are 8.8 and 9.1 g/100 for genomic selection, and 8.6 and 9.0 g/100 for phenotypic selection; for low and high seed oil contents (L.SOC and H.SOC) are 19.6 and 20.4% for genomic selection, and 19.0 and 20.0% for phenotypic selection. Numbers along the X-axis in each panel represent the number of lines retained under each selection regime

**Fig. 4:**
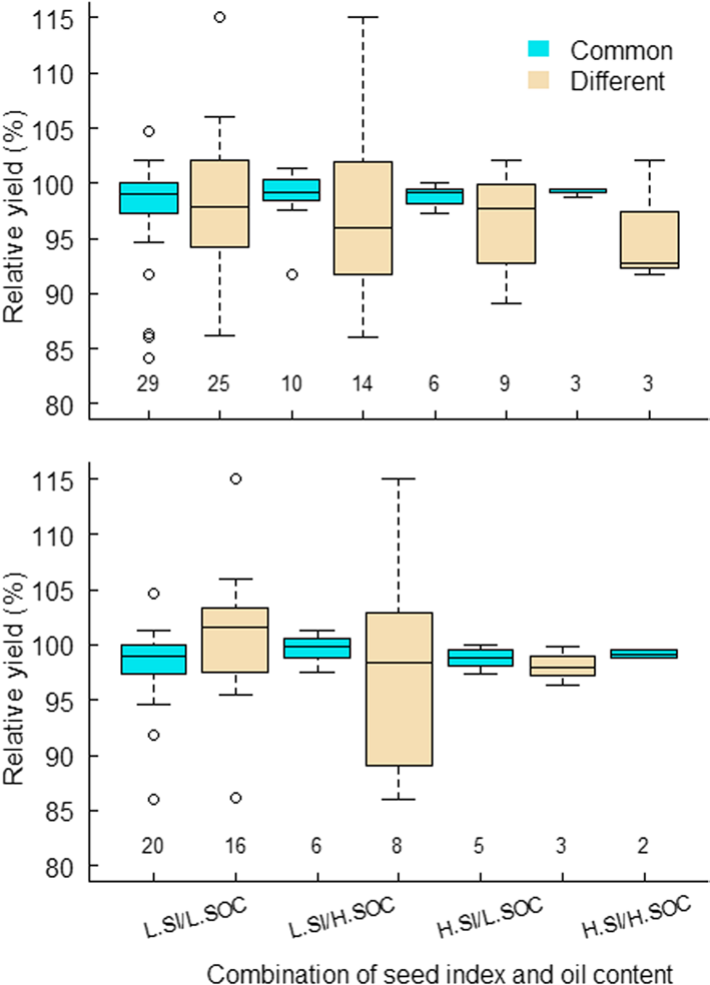
Relative lint yield variation for the number of RIL selected commonly and differently by genomic prediction of the 2016/17 and 2018/19 season phenotyping and phenotypic means of a pooled analysis under selection objective for simultaneous improvement of lint percentage, seed index and seed oil content. Relative lint yield was calculated against Sicot 71, the highest yielding parent (2372.2 kg/ha) based on a pooled analysis. Top and bottom panels represent selection scenarios for three seed yield traits under two levels of lint percentage, while combined with low and high seed index and seed oil content with truncation points referring to Fig. 3. Numbers along the X-axis represent the number of lines retained under each selection regime

Selection effectiveness based on GEBVs predicted from single-season phenotypic means was poor, when measured by the retained lines commonly kept by selection based on phenotypic means of a pooled analysis and also whether the best yielders were retained (Figs. S4 and S5). Applying the similar criteria in comparison, selection using GEBVs from any two-season phenotypic means became highly competitive to the one using GEBVs from three-season phenotypic means (Figs. 3 and 4; Figs. S6, S7, S8 and S9), and the latter was almost equivalent to selection based on overall phenotypic means (Figs. S8 and S9). Therefore, breeders could proceed selection decision based on GEBVs predicted from any two-season phenotyping. To further illustrate this finding, we chose GEBV-based selection from the 2016/17 and 2018/19 phenotypic means as an example. Compared to the GEBVs from the other paired season phenotypic means, GEBVs of this example season pair exhibited the weakest correlation with phenotypic means of a pooled analysis. Therefore, a proved case for genomic selection with this example should support the other two-season pairs in this study, while an expectation of its improved selection effectiveness.

Genomic selection kept 14–21% of the population (36–54), under the least restrictive scenarios; however, with stringent scenarios, the number of RILs kept reduced to around 15 or less (Table 5). The largest reduction was with the increased SI, followed with the increased SOC and the least for LP. The same phenomena were observed when selection was based on overall phenotypic means. Thus, the variations of SI and SOC in the population were the most limiting factors determining the number of lines retained. Nevertheless, both selection approaches showed competitiveness and effectiveness in identifying and capturing the individuals with the combinations of high values of all three traits despite being rare in the population. The retained individuals had LI ranging from 6.9 to 8.9 g/100 seeds with a mean of 8.0 g lint /100 seeds, which is within the range of the parents (Table S1), suggesting fibre productivity per seed was at least being maintained along with improved other fibre and seed traits.

Yield variation of selected populations under different scenarios are illustrated in Fig. 3. No matter what was used for selection, means of lint yield in selected populations did not change much when moving to higher SI or SOC or both, however, selection for higher SI always resulted in a reduced variation by excluding higher as well as lower yields. When GEBVs were used for selection, selection for higher SOC did not reduce the variation of lint yield although capturing more lines with low yields, particularly when the cut point for LP was set low. In contrast, when phenotypic results were used and selected for higher SOC, the variation of lint yield in the resultant populations was always reduced via excluding lower and higher yields. The greatest reduction of the variations for lint yield was in the scenarios where applying selection for higher LP as well as SI resulted in smaller numbers of lines with the highest yielders being only comparable to the best yielding parent, Sicot 71. In other words, many of the higher yielding RILs retained under the less stringent selection scenarios were all excluded. The evidence again highlights breeding challenges for combining high yield, high LP and high SI in cotton.

In the scenarios with the low selection cut point for SI, genomic selection was able to capture 42–56% of individuals the same as those retained by phenotypic selection (Fig. 4). In those with the high cut point for SI, this proportion varied from 40 to 100%. For lint yield potential, the groups commonly selected showed less variation but higher means than the ones selected differently by GEBVs and phenotypic means. On most occasions, highest yielding lines were only selected by GEBVs but not through the phenotypic results (Table S6).

As expected, GEBV-based selection for the other paired two-season phenotypes performed more comparable to phenotypic selection (Tables S4 and S5; Figs. S6 and S7). We conclude that selection based on GEBVs predicted from any two-season phenotypic results in this study could identify a good proportion of elite lines consistently selected by phenotypic selection, while could save the time and resource required in continuing field phenotyping of test population.

### Simulation studies for QTL mapping and genomic prediction

In QTL mapping, among 50 replicates, the LD-Bayes was able to detect four out of five major QTLs regardless of the effects to be common or season specific with high confidence (i.e. with frequencies to detect QTLs more than 0.5) (Table S7). The LD-Bayes was also able to detect two out of five QTLs with minor effects with high frequency. In contrast, both Bayes B and Bayes C were able to detect three out of the five major QTLs with high confidence, and they had a low power to detect any of the five minor QTLs. All the three Bayesian approaches showed equivalently strong ability to control the false positives (Table S7).

In regard to the GP, the LD-Bayes, Bayes B and Bayes C performed roughly equivalently well with LD-Bayes showed slightly better mean prediction accuracy over the 50 replicates (Fig. S10). These three methods all outperformed the BG-BLUP.

## Discussion

### Consensus of QTLs identified in this and other studies

The QTLs identified for four traits including LP, SI, LI and SOC in this study among Australian cotton varieties are often located in the same genomic regions previously reported for those traits. For example, all QTLs identified here for LP and SOC were at least reported in one previous study and are detailed below. There are only one novel QTL for SI and three for LI identified in this study. The consensus between this and other studies suggests A07, D01 and D13 are the common chromosomes for LP QTLs (Liu et al 2015a; Ma et al. 2018; Zhu et al 2021; Chen et al. 2022; Li et al. 2023) and A03 is also important (Wang et al. 2021; Li et al.^2023^). Of the three major LP QTLs, the signal regions covered by LD-223 and LD-1071 have been reported to be important in other populations (Gu et al. 2020; Zhu et al. 2021; Wang et al. 2021; Chen et al. 2022; Li et al. 2023). For SI QTLs, A08, D01 and D02 are the common chromosomes (Fang et al. 2017; Ma et al. 2018; Wang et al. 2021; Zhu et al. 2021). The genomic region of LD-1172 identified to be a major SI QTL (PVE = 0.06) in this study overlaps with the region identified to be associated with both SOC and LP by Hu et al. (2022). For SOC, A07, D02 and D08 are the common chromosomes (Shang et al. 2016; Liu et al. 2015b; Ma et al. 2019; Zhao et al. 2019; Hu et al. 2022). In addition, the genomic region of LD-157 with a PVE of 0.05 at A02 has been found to be associated with SOC in both *G. hirsutum* RIL and natural populations (Liu et al. 2015a; Hu et al. 2022), and the QTL LD-1426 with a PVE of 0.04 was also identified at least in two previous association studies (Liu et al. 2015b; Ma et al. 2019). The same applies to the LI QTL LD-1106 (Ma et al. 2018; Wang et al. 2021).

The above consensus for the QTLs identified in this study suggests some major and stable QTLs deserve further detailed dissection. They include LP QTLs LD-223 at A03 and LD-1071 at D01, that each captured ≥ 0.08 PVE; SI QTLs (LD-1172, LD1225) on D02 together with a PVE of 0.10, and SOC QTLs LD-554 at A07 and LD-1178 at D02 with a PVE of 0.12 and 0.08, respectively (Table 2). The effort can be from fine mapping for underlying candidate genes, allelic interaction to haplotypes, like that reported elsewhere for LP (Chen et al. 2022), for SI (Liu et al. 2022), and for SOC (Liu et al. 2020a; Hu et al. 2022). When gene or molecular markers are identified, they can facilitate the application of marker-assisted breeding in cotton to assemble favourable alleles of the traits (for examples, Fang et al. 2017; Ma et al. 2018, 2019; Zhao et al. 2019; Zhu et al. 2021). The information can also be used to investigate haplotypes, their origin and prevalence in breeding germplasm. Breeders can use such information for selecting better diverse parents in the design and making of crosses. Subsequently, that would ensure further assorting and assembling of diverse and favourable alleles to boost desirable recombinants in breeding populations for target traits (see the last section in discussion).

### Merits of the QTL mapping approach developed in this study

One of the major goals of this work was to introduce a novel Bayesian regression method that can do both QTL mapping and genomic prediction. Conventional Bayesian high dimensional regression models often struggle with handling the correlation structure between the SNPs, and lost power to detect QTL when high LD is present in the data. As an improvement, our new Bayesian approach can account for the linkage disequilibrium (LD) structure among the SNPs by assigning a common variance for the effects of SNPs within a LD block, and by considering each LD block as a unit instead of each single SNP in the multiple hypothesis testing to judge QTL. This advantage of the LD-Bayes clearly reflected in the simulation study, where the LD-Bayes showed a considerably higher power to detect QTLs compared to Bayes B and Bayes C, especially for those minor QTLs with small genetic effects (Table S7).

Another drawback of MCMC-based Bayesian regression methods is their high computational cost, and they often become infeasible for an ultra-high-dimensional genomic data set with millions of SNPs. Our LD-based Bayesian method provides a possible solution to significantly reduce the computational cost of Bayesian regression. Before the statistical analysis, we can conduct a dimensional reduction on SNPs in each LD block using methods such as principal component analysis (Li et al. 2018) and use a few PCs which explain a large proportion of genetic variation to replace hundreds of SNPs in each LD block in the QTL or genomic prediction analyses. Hence, we expect that our method can efficiently analyse large scale GWAS or genomic prediction data even with thousands of individuals and millions of SNPs, which may become more and more common in the area of plant molecular breeding in the near future as sequencing or genotyping costs drop. However, note that since the size of our data set in the present study is moderate, the original SNP data were used in both QTL and genomic prediction analyses.

### How to overcome the challenges for breeding higher crop value in cotton

The motivation of this study is to find breeding strategies for maintaining or improving seed, oil yield under the on-going effort of breeding for higher lint yield in cotton so that they can further enhance the entire crop value and its environmental resilience. In the multiple-parent derived population examined in this study, we observed an undesirable and dominant influence from a single parent on the segregation and recombination within the population for two important lint and seed yield traits, i.e. LP and SI. Obviously, these are the key underlying factors reducing the presence of RILs with desirable combinations of lint, seed and oil yields (Table 5). This situation becomes worse, with the unfavourable relationships of LP with SI and SOC, respectively, despite it being only moderately strong (Table 3). The results of retrospective selection in this study provide further evidence and suggest that the negative relations of LP and SI are much more influential than expected (Table 5). Nevertheless, as long as breeders can strike a good balance between LP and SI, there is no major challenge for maintaining or improving seed oil content or oil yield, because there is a positive relationship between SI and SOC, and SOC’s negative relation with LP is much weaker.

The inverse relation between LP and SI observed in this study of Australian germplasm is consistent with the results of many previous studies, which reported correlation coefficients from − 0.24 to 0.63 (Fang et al. 2017; Liu et al. 2017; Wang et al. 2021; Zhu et al. 2021; Hu et al. 2022; Li et al. 2023). This range implies two factors: (1) The degree of negative relations of LP with SI varies significantly in different cotton germplasm, and (2) the relationship can deteriorate further under continuous selection pressure for higher LP. For example, recently released cotton varieties have lower SI (seed weight) than those released a decade ago, but have higher LP (Conaty and Constable 2020; Maeda et al. 2023). When breeding targets shift to try to increase the entire value and environmental resilience of cotton, breeding approaches for higher lint yield by selection for higher LP should ask two questions seriously: how far can current breeding strategies go before reduced cotton seed size or oil content has serious negative impacts on its biological role as a planting seed and its values in post-harvest processes; the other is, how can you ensure that the impacts of the negative relation of LP and seed size (weight) are minimised such that selection for one trait will not result in significantly compromising the other trait?

To address the first question, there is a need for defining the minimal seed sizes for cotton, and this may already be determined, in part, by limitations of the existing planting, ginning and, screening equipment’s ability to handle smaller seeds as well as the biological resilience of small seeded cotton in the field in the face of a changing climate. As we already appear to be approaching such limits this implies that any future lint yield gains from breeding will need to be smarter and focus simultaneously on multiple components of lint yield not just LP. There is a large variation of seed productivity within cotton germplasm, and evidently the medium-size seeds are reported in general to be more productive in lint than small or large ones (Minton and Supak 1980; Main et al. 2013), except where they have been selected for high LP. Seed productivity could be further improved by increasing fibre density on seeds or modifying seed development process (Ruan 2013; Clement et al. 2014; Liu et al. 2020b). As mentioned previously, as long as seeds stay within a reasonable size range, breeding for stabilising or improving seed oil should follow automatically, because of the positive relationship between SI and SOC reported in this study and many previous studies (Liu et al. 2015a, b; Shang et al. 2016; Hu et al. 2022). The above effort to select for different yield components concurrently should also lead to increased boll size, as seed and boll sizes are positively correlated (Wang et al. 2021; Zhu et al. 2021), and relatively large bolls can also benefit some fibre quality traits, including fibre length and micronaire (Ruan 2013; Liu et al. 2017). Therefore, breeding efforts for maintaining seed size and seed productivity together should help in the continuous gain in lint yield and fibre quality.

To address the second question, current breeding should emphasise more allelic variation or genetic diversity for the three traits of LP, SI and SOC. Traditional practices for increasing genetic diversity including selecting material using origin and pedigree, as well as phenotypic performance are still vital. However, screening for allelic or haplotypic variations for individual traits and combination in those consensual major and stable QTLs identified in this and other studies would provide more precise information on the true genetic make-up of seed traits in cotton germplasm developed from different times, regions or programmes and aid in the selection of parents for crosses. This can be done by following the various studies of fibre quality properties (Ma et al. 2018; Li et al. 2023), LP and seed quality characters (Ma et al. 2019; Chen et al. 2022; Liu et al. 2022) reported already. They also provide examples of how such variation is important to increase LP (Chen et al. 2022), SI (Liu et al. 2022) and SOC (Zhao et al. 2019) in cotton. For SI, Liu et al. (2022) reported the existence of a major QTL on A07, namely *GhSI7*, and its associated three haplotypes in *G. hirsutum*. Interestingly, only the combination of haplotype 2 with the others leads to increase in SI in the derived population. Despite the large SI difference of our four parents in this study, the SI distribution, and absence of a SI QTL on A07 may explain why all four parents do not possess haplotype 2 but may have the others (Tables 1 and 2). If this is the case, introducing novel SI haplotypes into our elite germplasm background could be useful for correcting the undesired and dominant influence of single parents on trait distributions observed in this study (Table 1) and may mitigate or eliminate the accumulative effect due to selection for higher LP on seed size, seed weight or oil content, i.e. low SI and SOC (Fig. 2).

When new haplotypes are identified, and molecular markers are available, marker-assisted breeding could be applied in a straightforward manner. However, given that the genomic regions identified here and potentially useful for tracking and selection are only responsible individually for a limited amount of the observed variation (e.g. 12% is the highest PVE in this study), the approach may not be cost-effective. As demonstrated in this study, an alternative is to integrate this information with genomic prediction on all the traits, which can be done using the same Bayesian regression approach for QTL mapping. It allows the capture of large and small allelic effects important to trait performance so that selection can be conducted to identify desired individuals. In this study, despite the prediction accuracy being moderate, the predictions based on any two-season phenotyping can be effectively used to identify best performing individuals with a moderate to high consensus with the outcomes from normal phenotypic selection of a pooled analysis (Table 4, Figs. 3 and 4; Figs. S6 and S7). This is particularly evident under less stringent selection scenarios. Therefore, when the approach is applied early with reduced selection intensity, it should be effective in enriching the population with individuals with improved combinations of higher lint percentage, seed index and seed oil that would warrant testing in the field for confirmation and further selection for lint yield. The results of selection effectiveness in this study should be taken with caution as they rely on the predictive models established from a small training population and we also chose a recursive approach to estimate GEBVs and then used them for selection.

The effectiveness of using genomic selection in routine breeding will rely on the accuracy of predictions. Evidently, there was an improved accuracy for the models when accumulated phenotyping and genotyping data were generated and used for model training and evaluation in this study. In real breeding world, this means phenotyping data collected from different test environments e.g. test locations and seasons, can be used to further develop and refine the models as well as ensure that the models will explicitly account for genotype × environment interactions (Crossa et al. 2021; Gong et al. 2022). Applying a robust and accurate genomic selection model would speed up the delivery of breeding efforts while substantially saving resources and time required in field phenotyping. For example, in our breeding programme, we grow and test almost 5000 breeding lines per season in the 1st stage of field experiments often without replicate (e.g. F_5_) (Conaty et al. 2022). Under genomic selection-enabled breeding, we can genotype all test lines prior to their field testing, and then use GEBVs firstly to select those with improved genetics. We can decide selection intensity based on the accuracy of GPs, available resource as well as other tools available to optimise field testing. The retained population with enriched genetic potential can be tested in more than one representative locations, using the designs, for example, partially replicated designs taking account of genetic relatedness and crop specific non-genetic and residual models (Cullis et al. 2006, 2020). After the first-season phenotyping, test line information can be added into the training population to further calibrate genomic prediction model and new GEBVs can be used for selection again. New GEBVs would be expected to be more accurate and reliable for selection decision, as demonstrated in this study; therefore, they would allow breeders to be confident of taking selected individuals into the latest stage of field test widely in breeding target environments by skipping the intermediate stage of field test.

The intricate inverse nature of the relationships between LP with SI and SOC and the favourable relation to SI and SOC have already been reported by many previous studies, which either observed them in structured genetic or natural populations (Ma et al. 2019; Zhao et al. 2019; Chen et al. 2022; Li et al. 2023) or in breeding germplasm (Zeng et al. 2015; Snider et al. 2016; Hu et al. 2022). Our phenotype network results suggest two likely causes: (1) sharing of genomic regions for QTLs of different traits and (2) the common regulation of different genes mapped in different genomic regions, either for the corresponding trait itself or from the other secondary traits (Table 2; Fig. 2). These suggest the involvement of a type of pleiotropic mechanism, i.e. common genes controlling the expression of different traits (Wang et al. 2021). Linkage and pleiotropic effects are commonly reported for cotton fibre yield and quality traits (Ma et al. 2018; Wang et al. 2021; Chen et al. 2022; Li et al. 2023). Introducing novel allelic variation is always helpful to manage such unfavourable genetic effects.

In cotton breeding practices, nowadays only LP out of the four traits studied here is routinely measured and used for selection, as it is important to lint yield. When shifting to breeding for the entire crop value, seed size will need to be measured and monitored as the part of the routine selection process. This change will certainly alleviate the accumulative selection effect for higher LP on SI and SOC via their negative association. However, its implementation and adaption will require the assistance of novel and rapid technologies for tracking seed samples and observing and recording seed weight and sizes. Advances in research and application of modern computer vision and image analysis should help in developing and deploying such technologies. When these phenotypic data are collected, it can be used for independent culling of poor lines or incorporated together with LP to estimate index traits for selection, for example, Seed-score (Bourland et al. 2022). More importantly, the data can be used for developing robust genomic prediction models for selection, which breeders can rely on to discard undesirable individuals before moving to testing in the field (Table 5).

## Conclusions

Inheritance and interrelation of four yield component traits which represent how harvestable yield translates into lint, seed and oil products in cotton production are examined in a multiple-parent-derived RIL population in this study. These yield traits and trait interrelations are all moderately heritable without any large influence of genotype × environment interaction. Despite being common in transgressive segregation in the population for individual traits, there was a low abundance for segregants with the desirable higher LP, SI and SOC. This is driven by the dominant influence of parental lines as well as the inverse relation of LP with both SI and SOC. A novel Bayesian linkage disequilibrium-based multiple-locus mixed model introduced in this study was proved to be better power for identifying QTLs and competitive for predicting breeding values of test individuals. It identified several stable and major QTLs for individual traits and revealed that some of them were anchored to the same genomic regions or exhibited their direct effect on a specific trait itself as well as directly or indirectly on some of the others, implying that different mechanisms cause the above negative interrelations. Genomic selection was shown to be as effective as phenotypic selection in capturing those individuals with improved lint, seed and seed oil traits, and when applied routinely, it should speed up developing new cotton varieties while saving the time and cost. However, the mechanism governing the inverse relation of LP and SI remains a key barrier for simultaneous improvement for lint, seed and oil yields and this challenge can be mitigated or managed when incorporating phenotyping for seed size in the breeding practice, identifying and introducing new allelic variation for seed traits in breeders’ elite germplasm, and developing and applying modern genomic selections.

### Supplementary Information

Below is the link to the electronic supplementary material.

Supplementary file 1 (PDF 1240 kb)

Supplementary file 2 (PDF 357 kb)

## Data Availability

The phenotype, genotype as well as the R code to implement the Bayesian regression methods will be publicly available in the CSIRO Data Access Portal (https://data.csiro.au/collection/62567) upon acceptance of the manuscript.
