# Supplementary material for: Quantitative genomics-enabled selection for simultaneous improvement of lint yield and seed traits in cotton (Gossypium hirsutum L.)
Source: Theor Appl Genet. 2024 May 26;137(6):142. doi: 10.1007/s00122-024-04645-6 (PMC11128407; doi:10.1007/s00122-024-04645-6)
Supplement: Supplementary file 1 — (PDF 1240 kb) [file 122_2024_4645_MOESM1_ESM.pdf]

# Supplementary file 1

## Quantitative genomics-enabled selection for simultaneous improvement of lint yield and seed traits in cotton (*Gossypium hirsutum* L.)

Zitong Li[1], Qian-Hao Zhu[1], Philippe Moncuquet[1], Iain Wilson[1], Danny Llewellyn[1], Warwick Stiller[2], Shiming Liu[2]

[1]CSIRO Agriculture and Food, Canberra, ACT 2601, Australia

[2]CSIRO Agriculture and Food, Narrabri, NSW 2390, Australia

**Table S1** Lint, seed yield traits and oil content of the four parental lines of the MAGIC RIL population

| Name | Release year | Leaf type | Lint percentage (%) | Seed index (g/100) | Lint index (g/100) | Seed oil content (%) |
|---|---|---|---|---|---|---|
| Sicot 71 | 2003 | Normal | 41.8 | 9.6 | 8.6 | 26.2 |
| Sicot F-1 | 2005 | Normal | 37.9 | 9.1 | 6.7 | 23.6 |
| Sicot 75 | 2008 | Normal | 42.4 | 8.4 | 7.6 | 19.7 |
| Siokra 24 | 2005 | Okra | 38.5 | 10.3 | 7.9 | 25.5 |

**Table S2** Summary of QTLs, their locations on the genome and their effects

| Chromosome | LD block | SNP_ID | Genomic positions (kb) | Size of genetic effects | Type of genetic effects |
|---|---|---|---|---|---|
| A01 | 20 | 46 | 4635 | Sampled from N(5, 2.25) | Common effect |
| A01 | 63 | 231 | 49377 | | Common effect |
| A02 | 158 | 589 | 71187 | | Common effect |
| A03 | 201 | 705 | 4588 | | G×E effect (first season) |
| A07 | 510 | 1592 | 326 | | G×E effect (second season) |
| A05 | 415 | * | 39356- 47889 | Sampled from N(1.5, 0.25) | Common effect |
| A08 | 608 | * | 90100- 91605 | | Common effect |
| D01 | 1147 | * | 62164- 62588 | | Common effect |
| D08 | 1666 | * | 46972- 47868 | | G×E effect (first season) |
| D10 | 1791 | * | 941- 1803 | | G×E effect (second season) |

* For each of these 5 LD blocks, effects were simulated at 10 randomly selected SNPs within the LD block.

**Table S3** Distribution of LD blocks in each chromosome

| Chromosome | Number of LD blocks |
|---|---|
| A01 | 115 |
| A02 | 61 |
| A03 | 102 |
| A04 | 57 |
| A05 | 113 |
| A06 | 61 |
| A07 | 47 |
| A08 | 82 |
| A09 | 85 |
| A10 | 61 |
| A11 | 81 |
| A12 | 73 |
| A13 | 119 |
| D01 | 97 |
| D02 | 108 |
| D03 | 65 |
| D04 | 49 |
| D05 | 110 |
| D06 | 56 |
| D07 | 81 |
| D08 | 96 |
| D09 | 70 |
| D10 | 50 |
| D11 | 52 |
| D12 | 85 |
| D13 | 72 |

**Table S4** Number of RIL lines retained when selecting for three yield traits sequentially based on genomic predictions of the 2016/17 and 2017/18 season phenotypic means and also phenotypic means of a pooled analysis of three season experiments

| Truncation value for sequential selection of | | | Number of lines retained | Reduced number of the lines by selection for | | |
|---|---|---|---|---|---|---|
| Lint percentage (%) | Seed index (g/100 seeds) | Seed oil content (%) | | Lint percentage | Seed index | Seed oil content |
| Genomic predictions[a] | | | | | | |
| 41.3 | 8.6 | 18.4 | 45 (17.6) | | | |
| 41.3 | 8.6 | 19.2 | 20 (7.8) | | | -25 |
| 41.3 | 8.9 | 18.4 | 12 (4.7) | | -33 | |
| 41.3 | 8.9 | 19.2 | 6 (2.3) | | -14 | -6 |
| | | | | | | |
| 42.0 | 8.6 | 18.4 | 28 (10.9) | -17 | | |
| 42.0 | 8.6 | 19.2 | 20 (7.8) | 0 | | -8 |
| 42.0 | 8.9 | 18.4 | 12 (4.7) | 2 | -14 | |
| 42.0 | 8.9 | 19.2 | 6 (2.3) | -0 | -14 | -8 |
| Phenotypic means | | | | | | |
| 41.0 | 8.6 | 19.0 | 48 (18.8) | | | |
| 41.0 | 8.6 | 20.0 | 20 (7.8) | | | -24 |
| 41.0 | 9.0 | 19.0 | 14 (5.5) | | -34 | |
| 41.0 | 9.0 | 20.0 | 6 (2.3) | | -14 | -8 |
| | | | | | | |
| 42.0 | 8.6 | 19.0 | 34 (13.3) | -14 | | |
| 42.0 | 8.6 | 20.0 | 13 (5.1) | -7 | | -21 |
| 42.0 | 9.0 | 19.0 | 10 (3.9) | -4 | -24 | |
| 42.0 | 9.0 | 20.0 | 5 (2.0) | -1 | -7 | -5 |

[a] Selection truncation points for LP, SI and SOC of genomic selection were determined by applying regression equation for predicted value and phenotypic results presented in Fig. S2.
Value in bracket represents the proportion of test lines kept from the population (%).

**Table S5** Number of RIL lines retained when selecting for three yield traits sequentially based on genomic predictions of the 2017/18 and 2018/19 season phenotypic means and also phenotypic means of a pooled analysis of three season experiments

| Truncation value for sequential selection of | | | Number of lines retained | Reduced number of the lines by selection for | | |
|---|---|---|---|---|---|---|
| Lint percentage (%) | Seed index (g/100 seeds) | Seed oil content (%) | | Lint percentage | Seed index | Seed oil content |
| Genomic prediction [a] | | | | | | |
| 42.6 | 8.2 | 19.1 | 53 (20.7) | | | |
| 42.6 | 8.2 | 19.9 | 18 (7.0) | | | -35 |
| 42.6 | 8.5 | 19.1 | 18 (7.0) | | -35 | |
| 42.6 | 8.5 | 19.9 | 6 (2.3) | | -12 | -12 |
| | | | | | | |
| 43.5 | 8.2 | 19.1 | 35 (13.7) | -17 | | |
| 43.5 | 8.2 | 19.9 | 10 (3.9) | -8 | | -25 |
| 43.5 | 8.5 | 19.1 | 12 (4.7) | -6 | -23 | |
| 43.5 | 8.5 | 19.9 | 4 (1.6) | -2 | -8 | -8 |
| Phenotypic means | | | | | | |
| 41.0 | 8.6 | 19.0 | 48 (18.8) | | | |
| 41.0 | 8.6 | 20.0 | 20 (7.8) | | | -24 |
| 41.0 | 9.0 | 19.0 | 14 (5.5) | | -34 | |
| 41.0 | 9.0 | 20.0 | 6 (2.3) | | -14 | -8 |
| | | | | | | |
| 42.0 | 8.6 | 19.0 | 34 (13.3) | -14 | | |
| 42.0 | 8.6 | 20.0 | 13 (5.1) | -7 | | -21 |
| 42.0 | 9.0 | 19.0 | 10 (3.9) | -4 | -24 | |
| 42.0 | 9.0 | 20.0 | 5 (2.0) | -1 | -7 | -5 |

[a] Selection truncation points for LP, SI and SOC of genomic selection were determined by applying regression equation for predicted value and phenotypic results presented in Fig. S2.
Value in bracket represents the proportion of test lines kept from the population (%).

**Table S6** Mean and range of relative lint yield for RIL lines retained after selecting for three yield traits sequentially based on phenotypic means of a pooled analysis of three season experiments and genomic predictions of the 2016/17 and 2018/19 season phenotypic means

| Selection criteria [a] | Phenotypic selection | | | GEBVs selection | | |
|---|---|---|---|---|---|---|
| | Mean | Min | Max | Mean | Min | Max |
| Low LP-Low SI-Low SOC | 97 | 84 | 113 | 98 | 84 | 115 |
| Low LP-Low SI-High SOC | 98 | 84 | 110 | 97 | 86 | 115 |
| Low LP-High SI-Low SOC | 96 | 89 | 101 | 97 | 89 | 102 |
| Low LP-High SI-High SOC | 99 | 96 | 101 | 97 | 92 | 102 |
| High LP-Low SI-Low SOC | 99 | 86 | 113 | 99 | 86 | 115 |
| High LP-Low SI-High SOC | 100 | 95 | 110 | 99 | 86 | 115 |
| High LP-High SI-Low SOC | 98 | 90 | 101 | 98 | 96 | 100 |
| HIgh LP-High SI-High SOC | 99 | 98 | 101 | 99 | 99 | 100 |

[a] The truncation values for lint percentage (LP), seed index (SI) and seed oil content (SOC) refer to Table 5.

**Table S7** The frequency of each QTL being deleted using methods including LD-Bayes, Bayes B and Bayes C over 50 replicates

| QTL | Model | | |
|---|---|---|---|
| | LD-Bayes | Bayes B | Bayes C |
| QTL1 | **0.92** | 0.78 | 0.88 |
| QTL2 | **0.76** | 0.54 | 0.66 |
| QTL3 | **0.54** | 0.28 | 0.38 |
| QTL4 | **0.70** | 0.54 | 0.68 |
| QTL5 | 0.34 | 0.26 | **0.44** |
| QTL6 | **0.76** | 0.24 | 0.32 |
| QTL7 | **0.36** | 0.18 | 0.22 |
| QTL8 | **0.38** | 0.16 | 0.24 |
| QTL9 | **0.44** | 0.14 | 0.22 |
| QTL10 | **0.54** | 0.16 | 0.26 |
| No. of false positives | 0.08 | 0.08 | 0.08 |

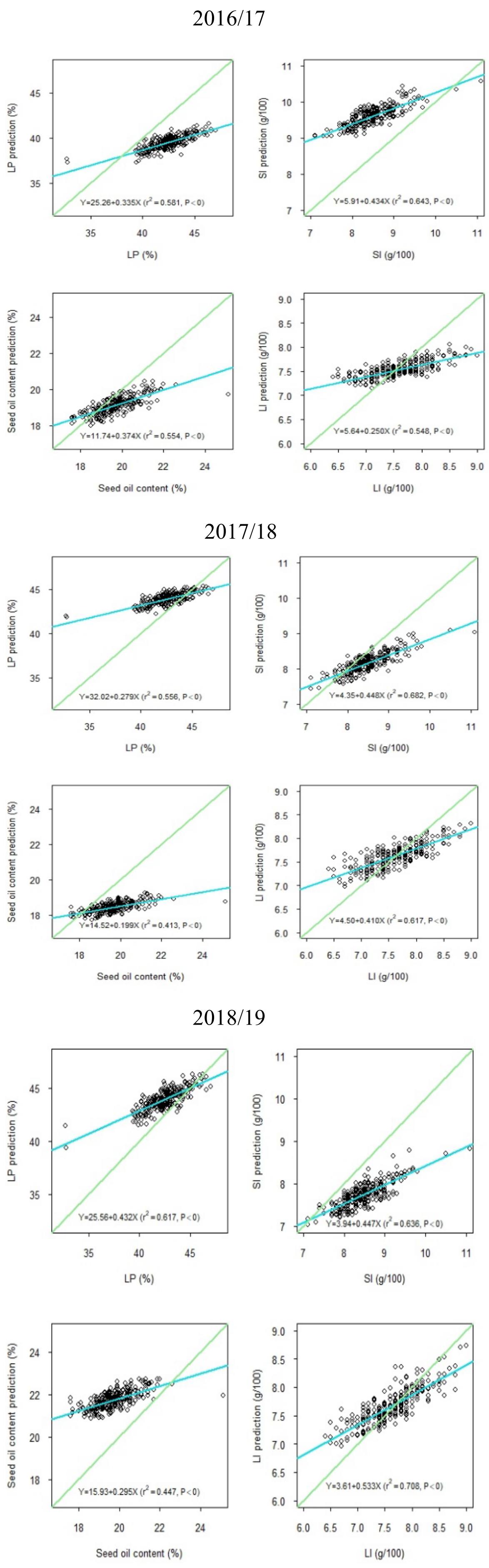


**Fig. S1** Relationship of RIL phenotypic means (x) of a pooled analysis of three season experiments and genomic predictions (y) based on single season phenotyping of lint percentage (LP), seed index (SI), seed oil content and lint index (LI) of a MAGIC population.

Light blue line represents linear regression of phenotypic means with genomic predictions; Green line represents linear regression where phenotypic means are equal to genomic predictions.

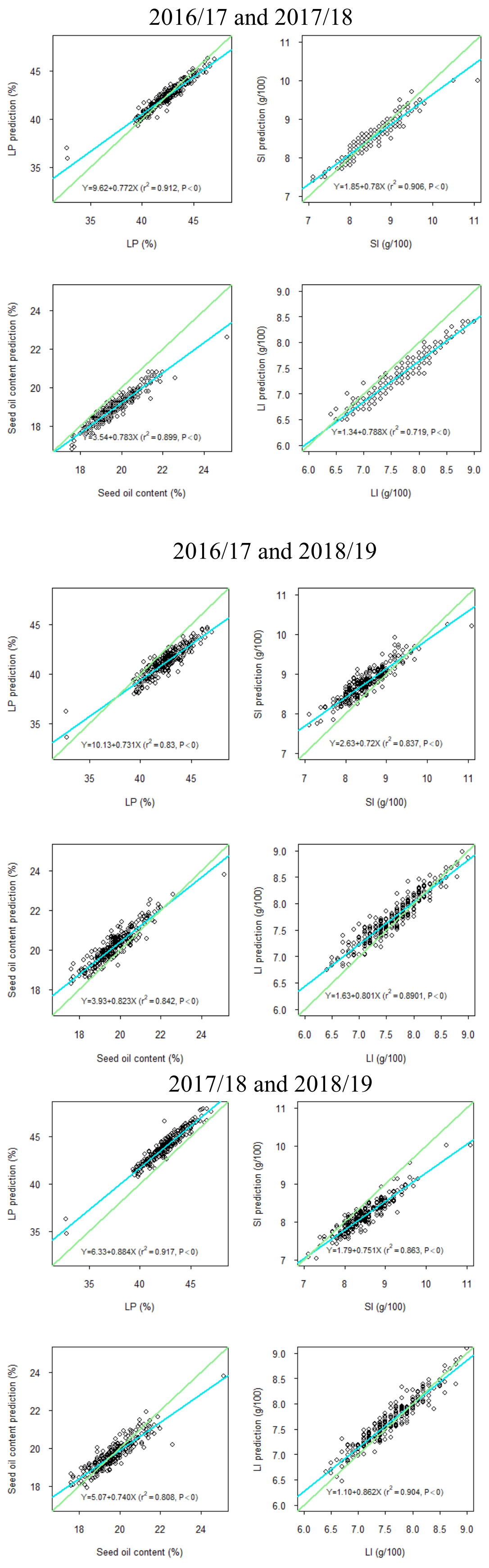


**Fig. S2** Relationship of RIL phenotypic means (x) of a pooled analysis of three season experiments and genomic predictions (y) based on paired two season phenotyping of lint percentage (LP), seed index (SI), seed oil content and lint index (LI) of a MAGIC population.

Light blue line represents linear regression of phenotypic results with genomic predictions; Green line represents linear regression where phenotypic means are equal to genomic predictions.

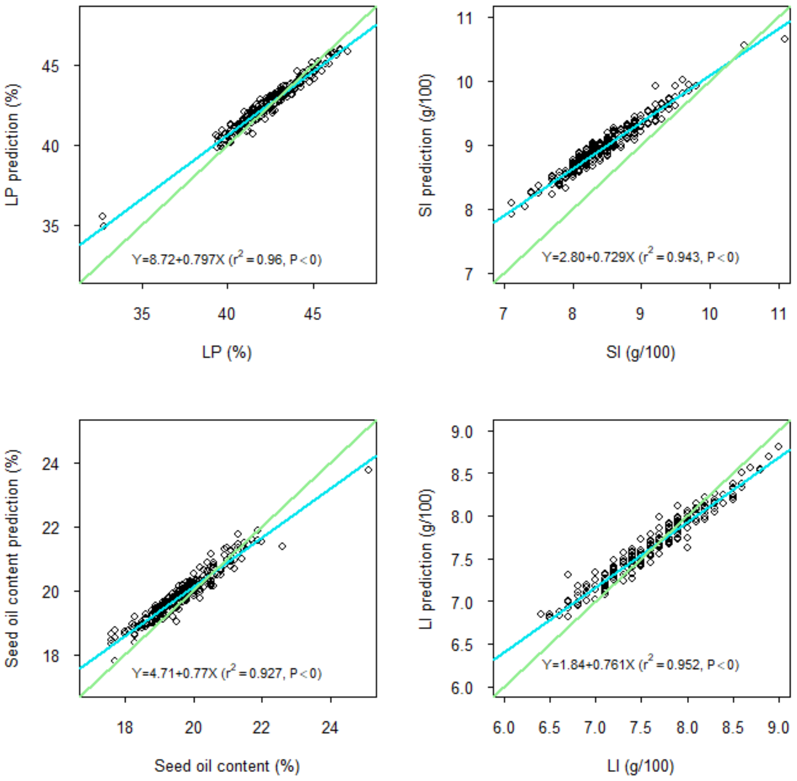


**Fig. S3** Relationship of RIL phenotypic means (x) of a pooled analysis of three season experiments and genomic predictions (y) based on three season phenotyping of lint percentage (LP), seed index (SI), seed oil content and lint index (LI) of a MAGIC population.

Light blue line represents linear regression of phenotypic results with genomic predictions; Green line represents linear regression where phenotypic means are equal to genomic predictions.

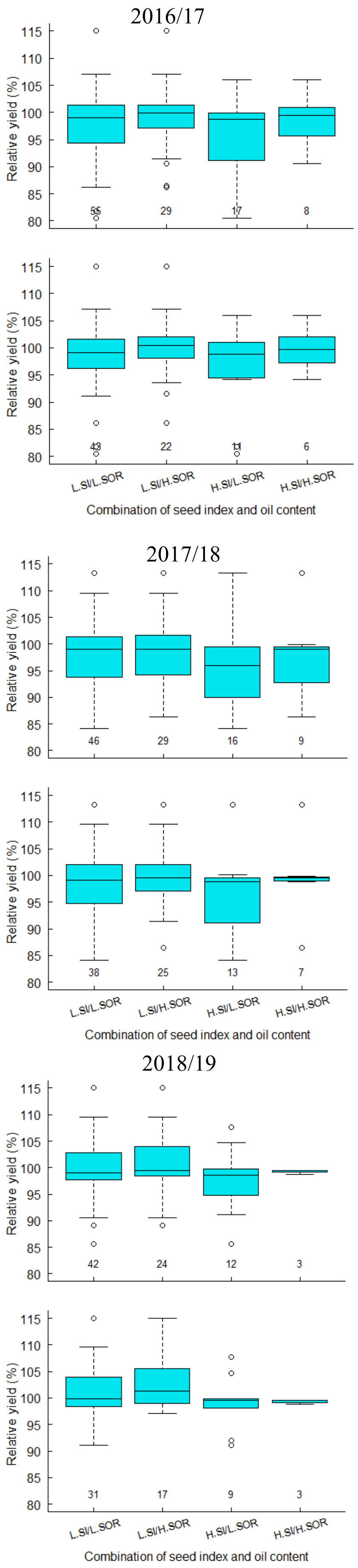


**Fig. S4** Relative lint yield variation of the retained RIL lines after simultaneous selection for lint percentage, seed index and seed oil content based on genomic predictions of single season phenotype.

Relative lint yield was calculated against Sicot 71, the highest yielding parent (2372.2 kg/ha) based on a pooled analysis.

Top and bottom panels represent selection scenarios for three seed yield traits under low and high lint percentage, while maintaining low and high seed index and seed oil content in combinations.

Truncation points for genomic selection are as follows:

In 2016/17, 39.0% (top) and 39.3% (bottom) for low and high lint percentage; 9.6 and 9.8 g/100 for low and high seed index (L.SI and H.SI); 18.8 and 19.2 % for low and high seed oil content (L.SOC and H.SOC).

In 2017/18, 43.5% (top) and 43.7% (bottom) for low and high lint percentage; 8.2 and 8.4 g/100 for low and high seed index (L.SI and H.SI); 18.30 and 18.5 % for low and high seed oil content (L.SOC and H.SOC).

In 2018/19, 43.3% (top) and 43.7% (bottom) for low and high lint percentage; 7.8 and 8.0 g/100 for low and high seed index (L.SI and H.SI); 21.5 and 21.8 % for low and high seed oil content (L.SOC and H.SOC).

Truncation points and selection results based on phenotypic means of a pooled analysis of three season experiments referring to the right panel in Fig. 3 or Fig. S8.

Numbers along the X-axis show the number of individual lines retained under respective selection regime.

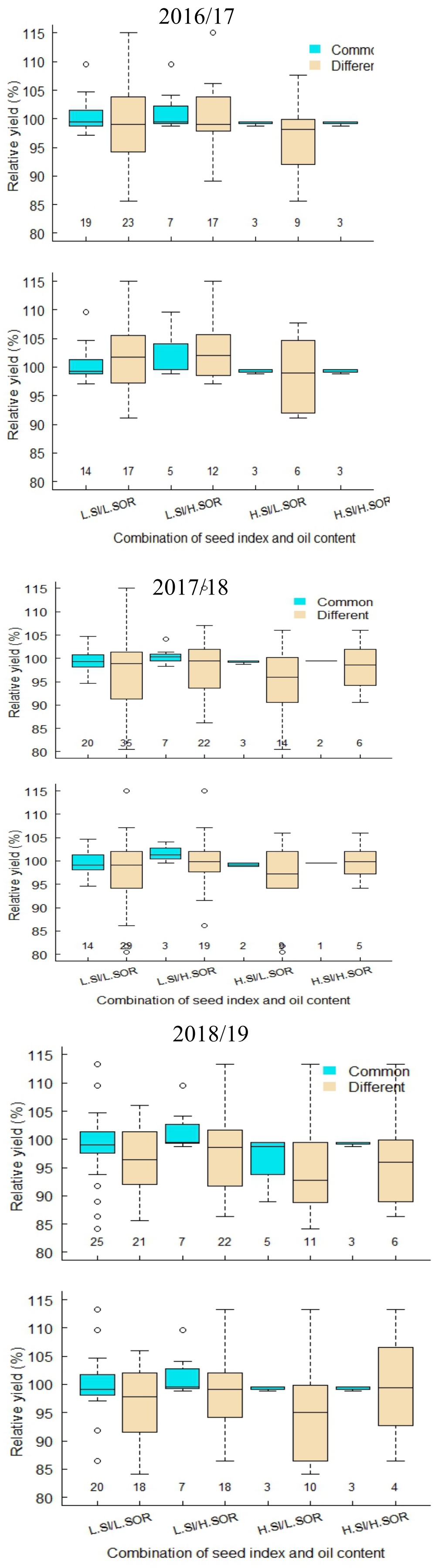


**Fig. S5** Relative lint yield variation for the number of RIL selected commonly and differently by genomic predictions of single season phenotype and phenotypic means of a pooled analysis of three season experiments for simultaneous improvement of lint percentage, seed index and seed oil content.

Relative lint yield was calculated against Sicot 71, the highest yielding parent (2372.2 kg/ha) based on a pooled analysis.

Top and bottom panel represent selection scenarios for three seed yield traits under low and high lint percentage, while maintaining low and high seed index and seed oil content in combinations with truncation points referring to Fig. S4.

Numbers along the X-axis show the number of individual lines retained under respective selection regime.

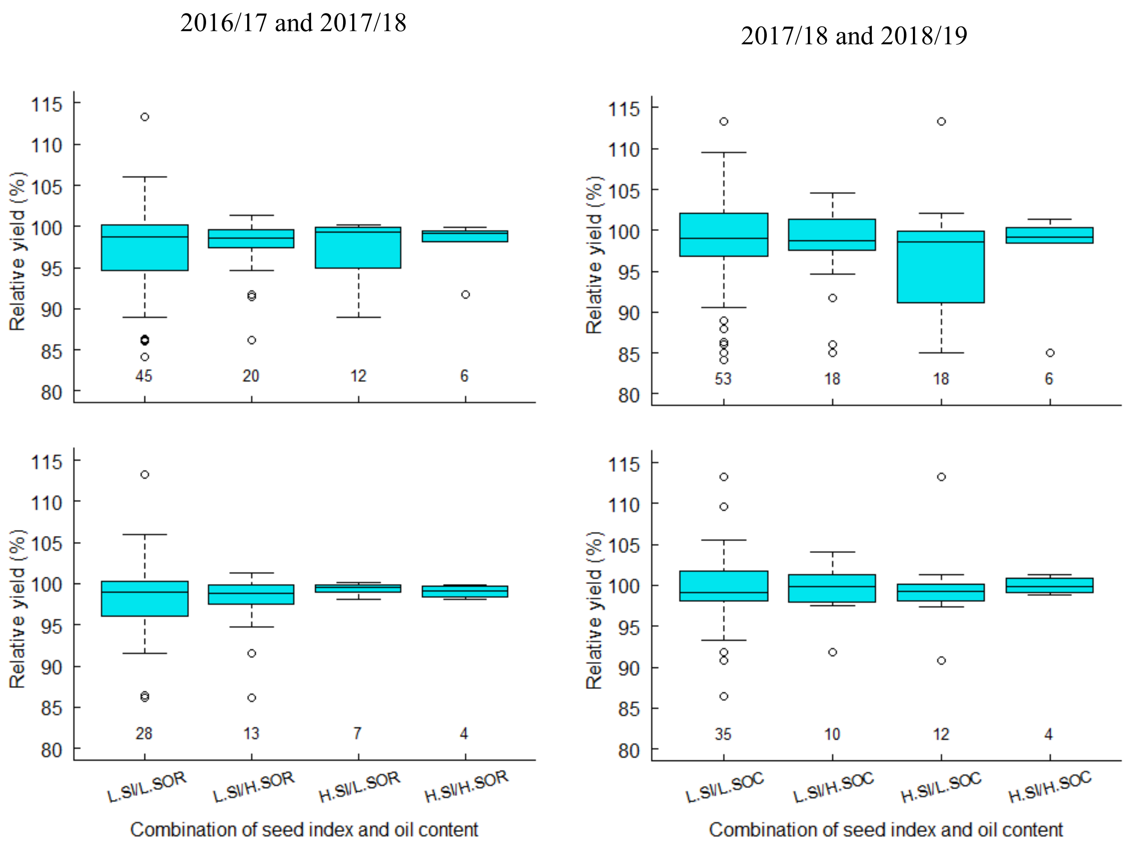


**Fig. S6** Relative lint yield variation of the retained RIL lines after simultaneous selection for lint percentage, seed index and seed oil content based on genomic predictions from paired two season phenotyping

Relative lint yield was calculated against Sicot 71, the highest yielding parent (2372.2 kg/ha) based on a pooled analysis.

Top and bottom panels represent selection scenarios for three seed yield traits under low and high of lint percentage, while maintaining low and high seed index and seed oil content in combinations.

Truncation points for genomic selection are as follows:

In the 2016/17 and 2017/18 season combination, 41.3% (top panel) and 42% (bottom panel) for low and high lint percentage; 8.6 and 8.9 g/100 for low and high seed index (L.SI and H.SI); 18.4 and 19.2 % for low and high seed oil content (L.SOC and H.SOC).

In the 2017/18 and 2018/19 season combination, 42.5% (top panel) and 43.5% (bottom panel) for low and high lint percentage; 8.2 and 8.5 g/100 for low and high seed index (L.SI and H.SI); 19.1% and 19.9 % for low and high seed oil content (L.SOC and H.SOC).

Truncation points and selection results based on phenotypic means of a pooled analysis of three season experiments referring to the right panel in Fig. 3 or Fig. S8.

Numbers along the X-axis show the number of individual lines retained under respective selection regime.

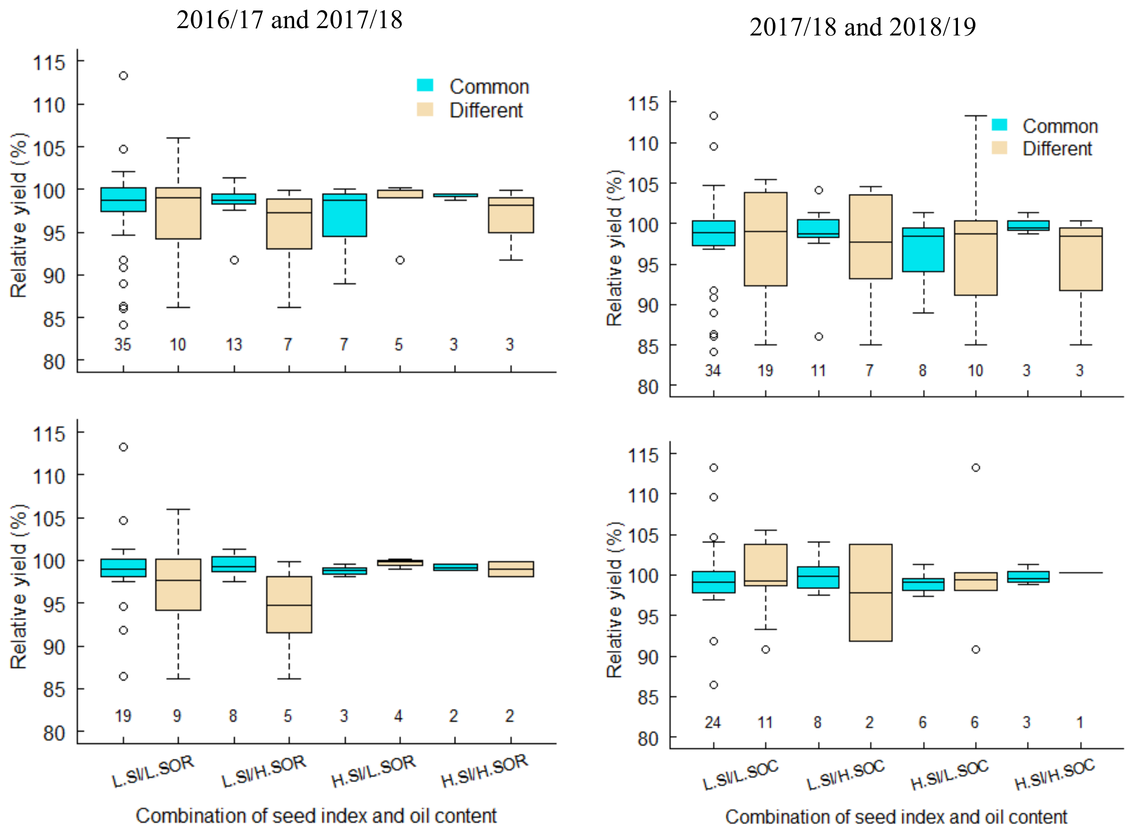


**Fig. S7** Relative lint yield variation for the number of RIL selected commonly and differently by genomic predictions based on the paired two season phenotyping and phenotypic means of a pooled analysis of three season experiments for simultaneous improvement of lint percentage, seed index and seed oil content.

Relative lint yield was calculated against Sicot 71, the highest yielding parent (2372.2 kg/ha) based on a pooled analysis.

Top and bottom panel represent selection scenarios for three seed yield traits under low and high lint percentage, while maintaining low and high seed index and seed oil content in combinations with the truncation points referring to Fig. S6.

Numbers along the X-axis show the number of individual lines retained under respective selection regime.

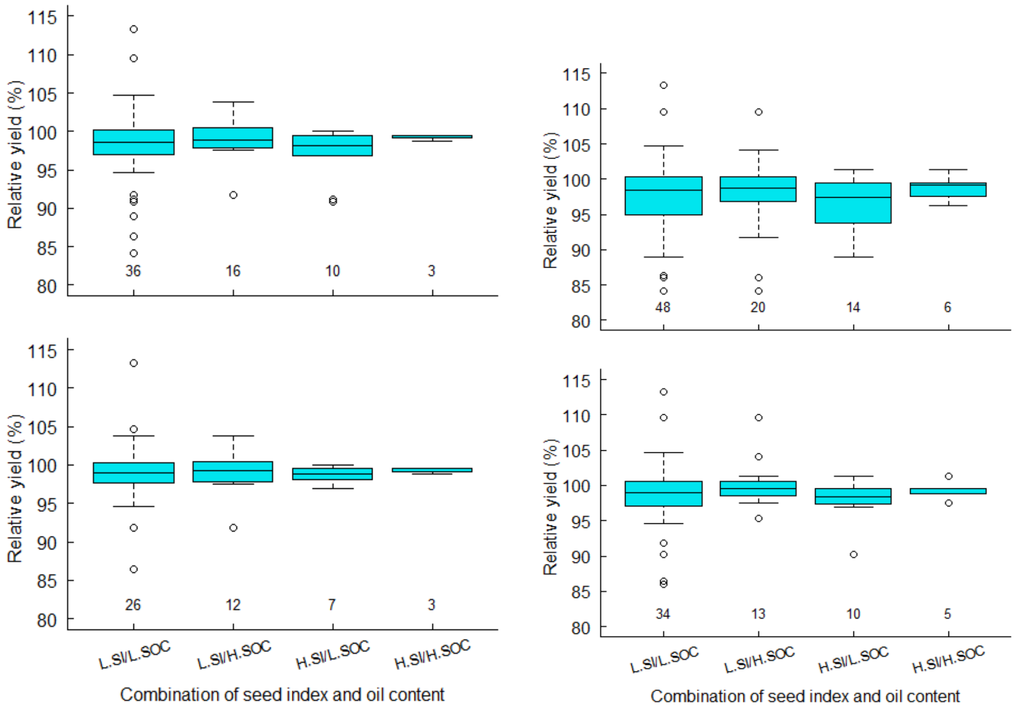


**Fig. S8** Relative lint yield variation of the retained RIL lines after simultaneous selection for lint percentage, seed index and seed oil content based on genomic predictions based on three season phenotyping (left panels) and phenotypic means of a pooled analysis of three season experiments (right panels).

Relative lint yield was calculated against Sicot 71, the highest yielding parent (2372.2 kg/ha) based on a pooled analysis.

Top and bottom panels represent selection scenarios for three seed yield traits under low and high lint percentage, while maintaining low and high seed index and seed oil content in combinations.

Truncation points for genomic selection are 41.3% (top panel, left) and 42% (bottom panel, left) for low and high lint percentage; 8.6 and 8.9 g/100 for low and high seed index (L.SI and H.SI) and 18.4% and 19.2 % for low and high seed oil content (L.SOC and H.SOC).

Truncation points for phenotypic selection are 41% (top panel, right) and 42% (bottom panel, right) for low and high lint percentage; 8.6 and 9.0 g/100 for low and high seed index (L.SI and H.SI); and 19.0% and 20.0% for low and high seed oil content (L.SOC and H.SOC).

Numbers along the X-axis show the number of individual lines retained under each selection regime.

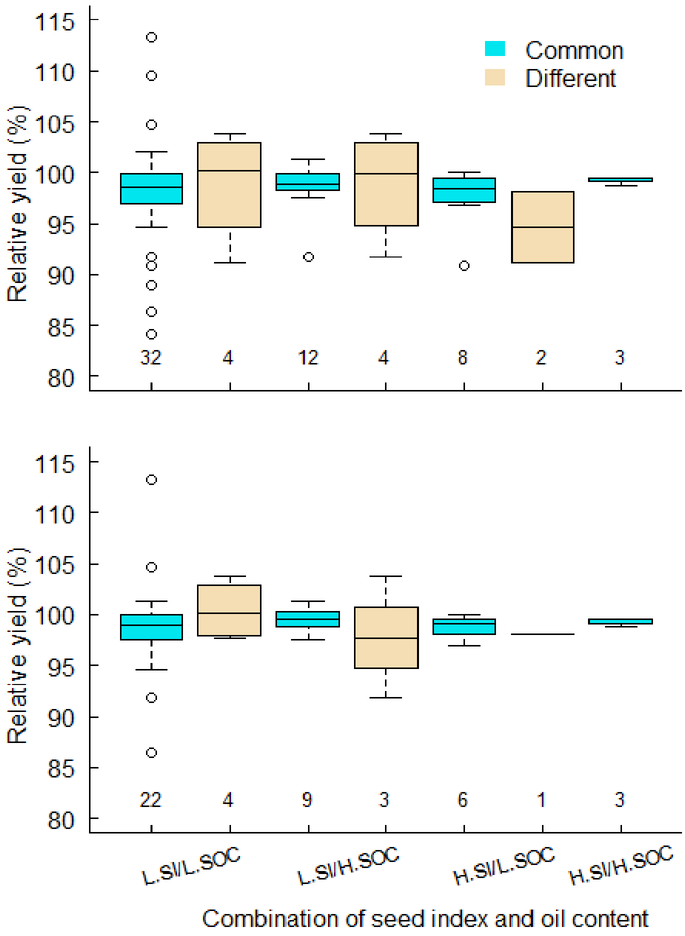


**Fig. S9** Relative lint yield variation for the number of RIL selected commonly and differently by genomic predictions based on three season phenotyping and phenotypic means of a pooled analysis of three season experiments for simultaneous improvement of lint percentage, seed index and seed oil content.

Relative lint yield was calculated against Sicot 71, the highest yielding parent (2372.2 kg/ha) based on a pooled analysis.

Top and bottom panel represent selection scenarios for three seed yield traits under low and high lint percentage, while maintaining low and high seed index and seed oil content in combinations with the truncation points referring to the notes in Fig. S8.

Numbers along the X-axis show the number of individual lines retained under respective selection regime.

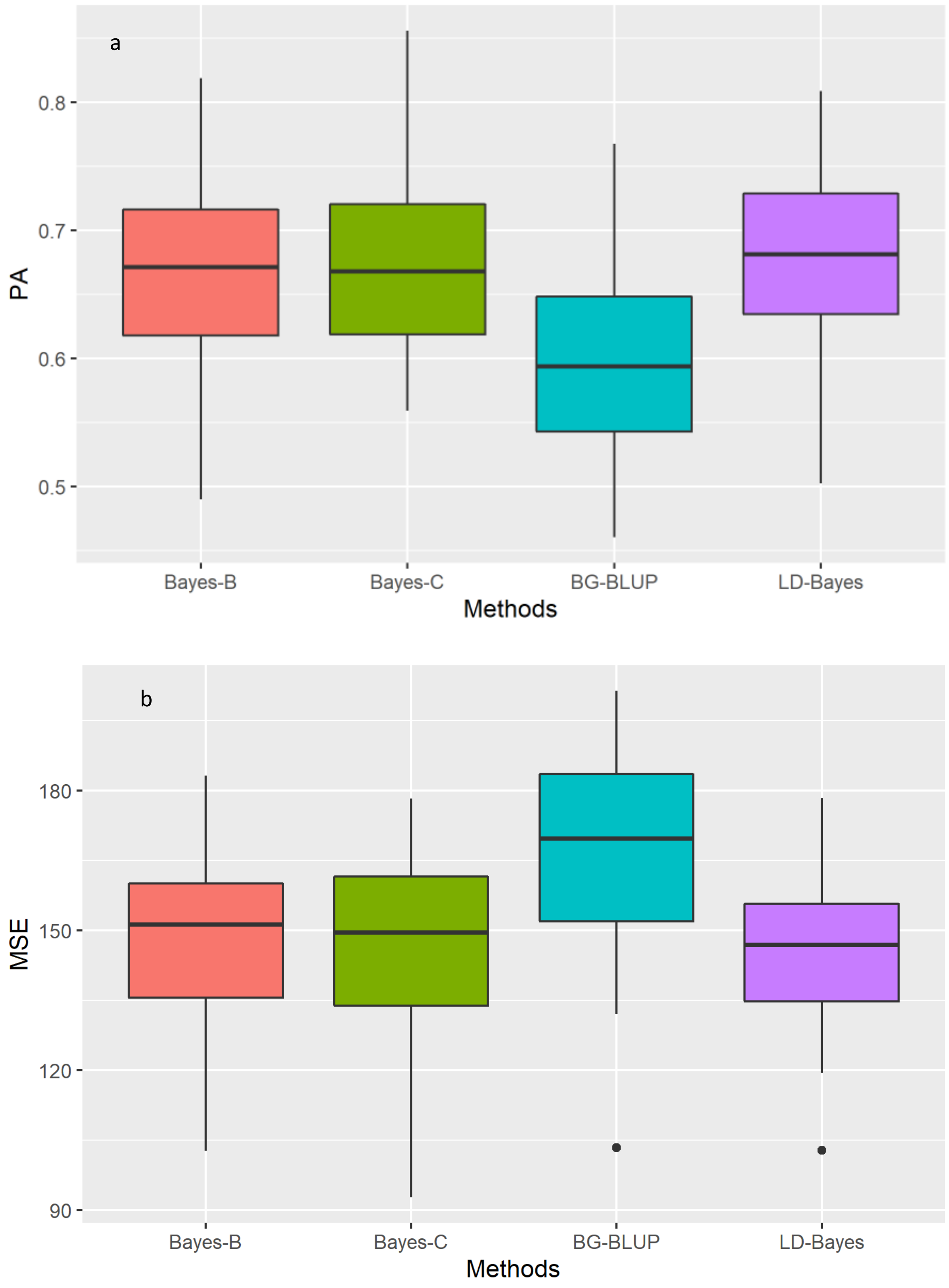


**Fig. S10** Boxplot of (a) Prediction accuracies (PA) and (b) Mean square error (MSE) of LD-Bayes, Bayes B, Bayes C and Bayes G-BLUP over 50 replicates.
