# Supplementary material for: Quantitative genomics-enabled selection for simultaneous improvement of lint yield and seed traits in cotton (Gossypium hirsutum L.)
Source: Theor Appl Genet. 2024 May 26;137(6):142. doi: 10.1007/s00122-024-04645-6 (PMC11128407; doi:10.1007/s00122-024-04645-6)
Supplement: Supplementary file 2 — (PDF 357 kb) [file 122_2024_4645_MOESM2_ESM.pdf]

Supplementary file 2

Quantitative genomics-enabled selection for simultaneous improvement of lint yield and seed traits in cotton (*Gossypium hirsutum* L.)

Zitong Li[1], Qian-Hao Zhu[1], Philippe Moncuquet[1], Iain Wilson[1], Danny Llewellyn[1], Warwick Stiller[2], Shiming Liu[2]

[1]CSIRO Agriculture and Food, Canberra, ACT 2601, Australia

[2]CSIRO Agriculture and Food, Narrabri, NSW 2390, Australia

1. The linkage disequilibrium Bayes model

$$y_{ij} = \beta_0 + \beta_{0j} + \alpha_{0i} + \sum_{k=1}^{p}\sum_{l=1}^{q_k} x_{ikl}\beta_{kl} + \sum_{j=1}^{m}\sum_{k=1}^{p}\sum_{l=1}^{q_k} x_{ijkl}\beta_{jkl} + e_{ij},\ e_{ij} \sim N(0, \sigma_0^2), \quad (1)$$

Equation (1) is equivalent to the following likelihood function:

$$P(y|\beta_0, \beta_{0j}, \alpha_{\mathrm{i}}, \beta_{kl}, \sigma_0^2) =$$
$$\prod_{j=1}^{m}\prod_{i=1}^{n}\frac{1}{\sqrt{2\,\pi\,\sigma_0^2}} exp\left(-\frac{\left(y_{ij}-\beta_0-\beta_{0j}-\alpha_{0\mathrm{i}}-\sum_{k=1}^{p}\sum_{l=1}^{q_k} x_{ikl}\beta_{kl}-\sum_{j=1}^{m}\sum_{k=1}^{p}\sum_{l=1}^{q_k} x_{ijkl}\beta_{jkl}\right)^2}{2\sigma_0^2}\right). \quad (2)$$

For simplicity, equation (2) is then re-written as

$$P(Y|\beta_0, \beta, \alpha_{\mathrm{i}}, \beta_{kl}, \sigma_0^2) = \prod_{i=1}^{N}\frac{1}{\sqrt{2\,\pi\,\sigma_0^2}} exp\left(-\frac{\left(\boldsymbol{Y}_i-\beta_0-W\beta-\alpha_{0\mathrm{i}}-\sum_{k=1}^{P}\sum_{l=1}^{q_k} X_{ikl}\mathrm{B}_{kl}\right)^2}{2\sigma_0^2}\right), \quad (3)$$

where

$$\boldsymbol{Y}_i = (y_{i1}, \dots, y_{im}),$$

$$W = \left.\begin{bmatrix} 1 & \cdots & 0 \\ \vdots & \ddots & \vdots \\ 0 & \cdots & 1 \end{bmatrix}\right\} m \times m,$$

$$\boldsymbol{\beta} = (\beta_{01}, \dots, \beta_{0m})^{\mathrm{T}},$$

The priors are

$$p(\beta_0) \propto 1_{(-\infty,\infty)}$$

$$p(\beta) \propto 1_{(-\infty,\infty)}$$

$$p(\alpha_{0i}) = \mathrm{N}(\alpha_{0i}|0, \sigma_\alpha^2)$$

$$p(\beta_{kl}) = (1-\gamma_{\mathrm{kl}})1_{\beta_{\mathrm{kl}}=0} + \gamma_{\mathrm{kl}}N(\beta_{kl}|0, \sigma_k^2)$$

$$p(\gamma_{\mathrm{kl}}) = \omega^{\gamma_{kl}}(1-\omega)^{\gamma_{kl}}$$

$$p(\omega) = \mathrm{Beta}(\omega|\mathrm{a}, \mathrm{b})$$

$$p(\sigma_0^2) = \mathrm{Inv}-\chi^2\left(\sigma_0^2\middle|\frac{a_0}{2}, \frac{b_0}{2}\right)$$

$$p(\sigma_\alpha^2) = Inv - \chi^2 \left(\sigma_\alpha^2 \middle| \frac{a_\alpha}{2}, \frac{b_\alpha}{2}\right)$$

$$p(\sigma_k^2) = Inv - \chi^2 \left(\sigma_k^2 \middle| \frac{a_k}{2}, \frac{b_k}{2}\right)$$

2. Full conditional distributions of model parameters:

The following full conditional distributions of model parameters are needed to derive the Gibbs sampling algorithm:

(i) The full conditional posterior of β_0: $p\left(\beta_0 \middle| \boldsymbol{\theta}_{-\beta_0}, Y\right) = \mathrm{MVN}\left(\beta_0 | \boldsymbol{\mu}_{\beta_0}, \boldsymbol{\Sigma}_{\beta_0}\right)$, where

$\boldsymbol{\mu}_{\boldsymbol{\beta}} = \frac{\boldsymbol{1}}{\boldsymbol{N}} \sum_{\boldsymbol{i=1}}^{\boldsymbol{N}} \left(\boldsymbol{Y}_i - W\beta - \alpha_i - \sum_{k=1}^{P} \sum_{l=1}^{q_k} x_{ikl} \beta_{kl}\right)$,

$\boldsymbol{\Sigma}_{\boldsymbol{\beta}} = N\sigma_0^2$.

(ii) The full conditional posterior of $\boldsymbol{\beta}$: $p\left(\boldsymbol{\beta} \middle| \boldsymbol{\theta}_{-\boldsymbol{\beta}}, Y\right) = \mathrm{MVN}\left(\boldsymbol{\beta} | \boldsymbol{\mu}_{\boldsymbol{\beta}}, \boldsymbol{\Sigma}_{\boldsymbol{\beta}}\right)$, where

$\boldsymbol{\mu}_{\boldsymbol{\beta}} = \frac{1}{N} (W^T W)^{-1} W^T \left(\boldsymbol{y}_i - \beta_0 - \alpha_i - \sum_{k=1}^{P} \sum_{l=1}^{q_k} x_{ikl} \beta_{kl}\right)$,

$\boldsymbol{\Sigma}_{\boldsymbol{\beta}} = n\sigma_0^2 (W^T W)^{-1}$.

(iii) The full conditional posterior of α_i: $p\left(\alpha_i \middle| \boldsymbol{\theta}_{-\alpha_i}, \boldsymbol{Y}\right) = \mathrm{N}\left(\alpha_i | \boldsymbol{\mu}_{\alpha_i}, \boldsymbol{\Sigma}_{\boldsymbol{\alpha}_i}\right)$ (for i=1,…,N), where

$$\boldsymbol{\mu}_{\alpha_i} = \frac{\sigma_\alpha^2}{\sigma_0^2} (\boldsymbol{y}_i - \beta_0 - W\beta - \sum_{k=1}^{P} \sum_{l=1}^{q_k} x_{ikl} \beta_{kl})$$

$$\boldsymbol{\Sigma}_{\boldsymbol{\alpha}_i} = \frac{\sigma_0^2}{\boldsymbol{m} + \frac{\sigma_0^2}{\sigma_\alpha^2}}$$

(iv) The full conditional posterior of B_{kl} and γ_{kl}: These two parameters are sampled together to guarantee the sampler is mixing well. Since $p\left(B_{kl}, \gamma_{kl} \middle| \theta_{-[B_{kl}, \gamma_{kl}]}, Y\right) = p(\gamma_{kl} | \theta_{-[B_{kl}, \gamma_{kl}]}, Y) p(B_{kl} | \gamma_{kl}, \theta_{-[B_{kl}, \gamma_{kl}]}, Y)$, the strategy is to first sample γ_{kl} from $p(\gamma_{kl} | \theta_{-[B_{kl}, \gamma_{kl}]}, Y)$, and then B_{kl} from $p(B_{kl} | \gamma_{kl}, \theta_{-[B_{kl}, \gamma_{kl}]}, Y)$ based on the latest update of γ_{kl}. The two distributions are derived as follows:

$p\left(\gamma_{kl} = 1 \middle| \theta_{-[B_{kl}, \gamma_{kl}]}, Y\right) = \frac{\omega E}{\omega E + 1 - \omega}$, where

$E = \frac{\sigma_{B_{kl}}}{\sigma_k} \exp\left(\frac{\boldsymbol{\Sigma}_{B_{kl}}}{2\sigma_{B_{kl}}^2}\right)$,

$$\boldsymbol{\Sigma}_{B_{kl}} = \frac{\sigma_{B_{kl}}^2}{\sigma_0^2} \sum_{\boldsymbol{i=1}}^{\boldsymbol{N}} X_{ikl} \left(\boldsymbol{Y}_i - \beta_0 - W\beta - \alpha_i - \sum_{s \neq l} \sum_{t \neq k} x_{ist} \beta_{st}\right),$$

$$\sigma_{B_{kl}}^2 = \frac{\sigma_0^2}{\sum_{\boldsymbol{i=1}}^{\boldsymbol{N}} X_{ikl}^2 + \frac{\sigma_0^2}{\sigma_\alpha^2}}$$

(v) The full conditional posterior of ω: $p(\omega) = \mathrm{Beta}(\omega|\mathrm{A}, \mathrm{B})$

$$A = \sum_{k=1}^{P} \sum_{l=1}^{q_k} \gamma_{kl} + a$$

$$B = \sum_{k=1}^{P} q_k - \sum_{k=1}^{P} \sum_{l=1}^{q_k} \gamma_{kl} + b$$

(vi) The full conditional posterior of σ_0^2 : $p(\sigma_0^2) = \mathrm{Inv} - \chi^2 \left(\sigma_0^2 \middle| \frac{A_0}{2}, \frac{B_0}{2}\right)$

$$A_0 = N + a_0$$

$$B_0 = (\boldsymbol{Y}_i - \beta_0 - W\beta - \sum_{k=1}^{P} \sum_{l=1}^{q_k} x_{ikl}\beta_{kl})^2 + b_0$$

(vii) The full conditional posterior of σ_α^2 : $p(\sigma_\alpha^2) = \mathrm{Inv} - \chi^2 \left(\sigma_\alpha^2 \middle| \frac{A_\alpha}{2}, \frac{B_\alpha}{2}\right)$

$$A_\alpha = m + a_\alpha$$

$$B_\alpha = \sum_{i=1}^{m} \alpha_i^2 + b_\alpha$$

(viii) The full conditional posterior of σ_k^2: $p(\sigma_k^2) = \mathrm{Inv} - \chi^2 \left(\sigma_k^2 \middle| \frac{A_k}{2}, \frac{B_k}{2}\right)$

$$A_k = \sum_{l=1}^{q_k} \gamma_{kl} + a_k$$

$$B_k = \sum_{l=1}^{q_k} \beta_{kl}^2 + b_k$$
